# Effectiveness of Chinese herbal medicine for cancer palliative care: overview of systematic reviews with meta-analyses

**DOI:** 10.1038/srep18111

**Published:** 2015-12-16

**Authors:** Vincent CH Chung, Xinyin Wu, Edwin P. Hui, Eric TC Ziea, Bacon FL Ng, Robin ST Ho, Kelvin KF Tsoi, Samuel YS Wong, Justin CY Wu

**Affiliations:** 1 Hong Kong Institute of Integrative Medicine, The Chinese University of Hong Kong, Hong Kong; 2 Jockey Club School of Public Health and Primary Care, The Chinese University of Hong Kong, Hong Kong; 3 Comprehensive Cancer Trials Unit, The Chinese University of Hong Kong, Hong Kong; 4 Chinese Medicine Department, Hong Kong Hospital Authority, Hong Kong; 5 Big Data Decision Analytics Research Centre, The Chinese University of Hong Kong, Hong Kong; 6 Department of Medicine & Therapeutics, The Chinese University of Hong Kong, Hong Kong

## Abstract

Chinese herbal medicines (CHM) are often used in managing cancer related symptoms but their effectiveness and safety is controversial. We conducted this overview of meta-analyses to summarize evidence on CHM for cancer palliative care. We included systematic reviews (SRs) with meta-analyses of CHM clinical trials on patients diagnosed with any type of cancer. Methodological quality of included meta-analyses was assessed with the Methodological Quality of Systematic Reviews (AMSTAR) Instrument. Fifty-one SRs with meta-analyses were included. They covered patients with lung (20 SRs), gastric (8 SRs), colorectal (6 SRs), liver (6 SRs), breast (2 SRs), cervical (1 SR), esophageal (1 SR), and nasopharyngeal (1 SR) cancers. Six SRs summarized evidence on various types of cancer. Methodological quality of included meta-analyses was not satisfactory. Overall, favorable therapeutic effects in improving quality of life among cancer patients have been reported. Conflicting evidence exists for the effectiveness of CHM in prolonging survival and in reducing chemotherapy and/or radiotherapy related toxicities. No serious adverse effects were reported in all included studies. Evidence indicated that CHM could be considered as an option for improving quality of life among patients receiving palliative care. It is unclear if CHM may increase survival, or reduce therapy related toxicities.

Defined as “total care” by the World Health Organization^1^, the primary aims of palliative cancer care are to promote adequate symptom control and to optimize quality of life before a “timely, dignified and peaceful death” in people with cancer^2^. Early, appropriate palliative cancer care can reach the goal of improving quality of life, increasing survival time, and reducing the need of aggressive care during the end of life^3^. Current model has shifted from confining palliative cancer care to the last 6 months of life to the whole disease trajectory^4^. Growing number of aging populations with increasing prevalence of cancer have made palliative care a global health priority^5^,^6^. While the role of palliative care is widely recognized, effective palliative care interventions are sparse^7^,^8^.

In view of limitations in conventional palliative care, the potential role of traditional Chinese medicine can be explored. It has long been used as a supportive intervention for cancer patients in China and other Asian countries^9^. It is also becoming popular in western countries in palliative cancer care^10^. As one of the major treatment modalities in traditional Chinese medicine, Chinese herbal medicine (CHM) has been widely used as adjuvant cancer treatment among Chinese communities^9^,^11^. Numerous systematic reviews (SRs) have been conducted to synthesize the effectiveness of CHM in palliative cancer care. One of the SRs published in English has synthesized evidence on the effectiveness of CHM on cancer symptom management. The results indicated mixed results for improving nutritional status, pain and quality of life but it is likely to be outdated^12^. There are also some reports on CHM’s effectiveness in reducing side effect of chemotherapy and in improving survival^13^.

We conducted an overview of meta-analysis to critically appraise and summarize clinical evidence on CHM for cancer palliative care. We aim to provide a broad overview on available evidence, which will inform clinicians, cancer patients and policy makers, and to identify methodological limitations of existing SRs so as to guide future research in this area.

## Results

### Characteristics of included SRs

**Figure 1:**
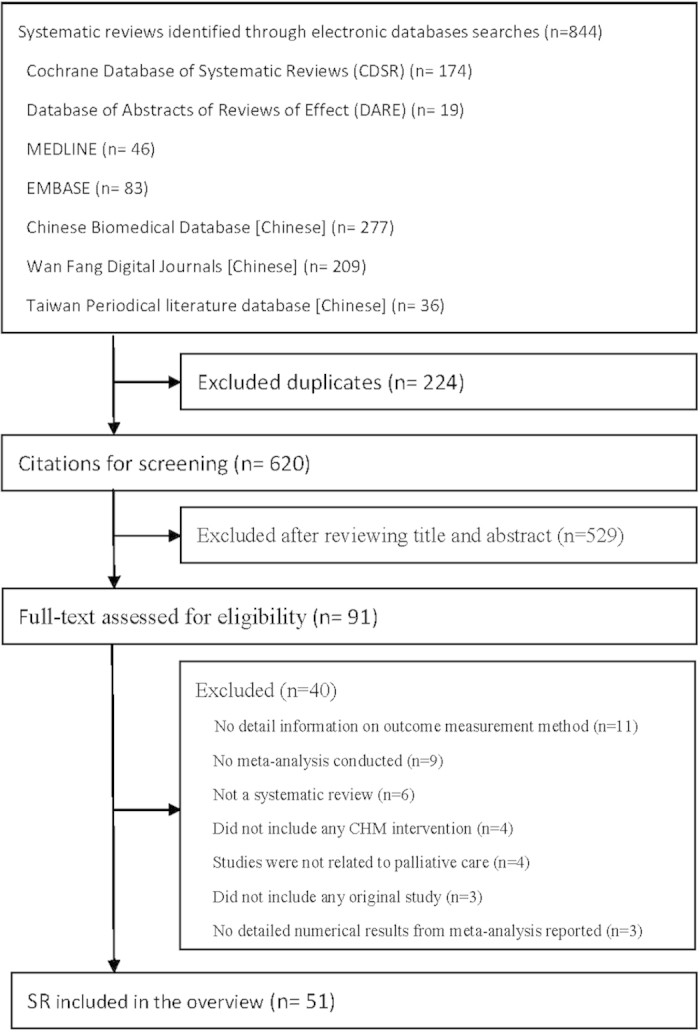
Flowchart of literature selection on meta-analyses of Chinese herbal medicines for cancer palliative care. Keys: CHM, Chinese herbal medicine; SR, systematic review.

A total of 844 citations were retrieved from the databases, among which 51 SRs met the inclusion criteria and were included in this overview (Fig. 1). The 51 included SRs were published between 2004 and 2014, with 34 (66.7%) published after 2012. Characteristics of these SRs can be found in Table 1. Eleven SRs (21.6%) were published in English and the remaining 40 (80.4%) were published in Chinese. Thirty-six SRs (70.6%) searched both international and Chinese databases and 30 of them (83.3%) reported the publication languages of included studies, with 24 (80.0%) only identified Chinese publications. Two (3.9%) were Cochrane SRs. Thirty-seven (72.5%) SRs only included randomized controlled trials (RCTs) while the remaining 14 (27.5%) included both RCTs and non-RCTs. Among 48 SRs that provided a cutoff date on literature search, 29 (60.4%) conducted literature search after 2011 with the most recent search conducted in January 2013. Lists of the included SRs were shown in Appendix 3.

Twenty (39.2%) SRs only included lung cancer patients, among which 19 SRs specifically focused on non-small cell lung cancer (NSCLC) patients. The remaining 31 SRs summarized evidence on patients with gastric (8 SRs), colorectal (6 SRs), liver (6 SRs), breast (2 SRs), cervical (1 SR), esophageal (1 SR), and nasopharyngeal (1 SR) cancers. Six SRs summarized evidence on various types of cancer.

All CHM interventions were used as an adjuvant therapy in the included SRs, with comparisons being CHM plus chemotherapy and/or radiotherapy versus chemotherapy and/or radiotherapy alone. Details on CHM evaluated can be found in Table 1. Twenty-eight SRs reviewed a single, specific type of CHM treatment, while the remaining 23 SRs summarized evidence on various types of CHM. Details of CHM reviewed in the 23 SRs were shown in Appendix 4.

Three types of outcomes were summarized among identified SRs, with 29 SRs provided results on quality of life (QoL), 23 SRs on survival rate and 11 SRs on chemotherapy and/or radiotherapy induced toxicity. These toxicities included leucopenia (9 SRs), nausea and vomiting (8 SRs), thrombocytopenia (6 SRs); anemia (5 SRs), neurotoxicity (2 SRs), diarrhea (1 SR), and stomatitis (1 SR). Details can be found in Table 1.

### Methodological quality of included SRs

Methodological quality of included SRs was shown in Table 2. Forty-nine (96.1%) SRs performed a comprehensive literature search. Forty-four (86.3%) SRs assessed and documented risk of bias among included studies. Thirty-nine (76.5%) SRs used appropriate methods to combine the findings. Nineteen (37.3%) SRs did not search for grey literature. Sixteen (31.4%) SRs provided the characteristics of included studies. Only two SRs (3.9%) provided a protocol. Although 39 (76.5%) SRs listed all included studies, only two (3.9%) provided a list of both included and excluded studies. No SRs stated conflict of interest for both the SR and the included studies. Nine (17.6%) stated conflict of interest for the SR itself, eight of which were published in English and only one was published in Chinese.

### Effectiveness of CHM for cancer palliative care

All included SRs used similar criteria for measuring the outcomes. QoL was assessed with the Karnofsky Performance Status (KPS) scale. A KPS score increment >10 score was defined as clinical improvement, while patients with KPS score increment >0 were considered as respondents. Survival was measured with survival rate across the follow up duration. Chemotherapy toxicities were measured by the World Health Organization toxicity criteria. Details on the effectiveness as well as quality of evidence of CHM for improving QoL and survival rate; and in reducing chemotherapy and/or radiotherapy related toxicities are shown in Tables 3–5. In the paragraphs below, abbreviation of the CHM is used if the SR focused specifically on one particular type of CHM. The general term of “CHM” is used if the SR provided evidence on various type of CHM.[Table t4]

### QoL

#### NSCLC

Fourteen SRs summarized evidence on CHM as an adjuvant intervention for improving QoL in NSCLC patients. These 14 SRs reviewed the effects of Compounds Kushen injection (KS) (3 SRs), Kanglaite injection (2 SRs), Shenqi Fuzheng injection (SFI) (2 SRs), Kang Ai injection (2 SRs), Zijinglong (1 SR), Xiaoaiping (1 SR), Shenfu injection (1 SR). Two reviewed mixed types of CHM. When compared to chemotherapy alone, combination of CHM and chemotherapy significantly improved QoL, as shown in the meta-analyses results. All but two meta-analyses showed homogeneity, with I^2^ values of 57.0% and 77.0% respectively.

#### Liver cancer

Three SRs summarized the add-on effects of CHM on QoL in liver cancer patients. One SR focused on KS and the other two reviewed a range of CHM. Meta-analyses from these three SRs showed that patients treated with KS or CHM plus transcatheter chemoembolization (TACE) had significantly greater improvement on QoL than those who received TACE alone. One SR also showed that CHM plus TACE provided a significantly higher increment on KPS score (pooled MD = 10.03, 95% CI = 8.98–11.07) than TACE alone. Nevertheless, high level of heterogeneity (I^2^ = 95%, p < 0.001) exist in this meta-analysis.

#### Gastric cancer

Two SRs reviewed the evidence of SFI plus chemotherapy for improving QoL among gastric cancer patients. Meta-analyses showed promising effect of SFI in both SRs. One which included TNM stage I-IV patients showed a significantly improved response (improvement rate, pooled RR = 3.14, 95% CI = 2.11–4.69). The other one which only included TNM stage III–IV patients demonstrated a significant, yet lesser effect (responder rate, pooled OR = 1.48, 95% CI = 1.26–1.57). Another SR which included various type of CHM found that, when compared to conventional care alone, combination of CHM and conventional care can slightly improve QoL score (pooled MD = 0.51, 95% CI = 0.21–1.82) in TNM stage III–IV patients. High heterogeneity (I^2^ = 86%, p < 0.01) was observed for this meta-analyses.

#### Colorectal cancer

Two SRs have reported add-on benefit of CHM in improving QoL. One Cochrane SR summarized evidence on advanced colorectal cancer patients, who are diagnosed as reaching stage IV in the American Joint Committee on Cancer Staging System or stage D with Union Internationale Contrele Cancer. The other SR did not provide tumor stage of included patients.

#### Breast cancer

Two SRs reported that, when compared to chemotherapy alone, KS plus chemotherapy significantly improved QoL in breast cancer patients.

#### Nasopharyngeal cancer

One SR summarized evidence of various CHM for improving QoL in patients with nasopharyngeal cancer. Meta-analysis showed that, the combination of CHM and radiotherapy led to a slight improvement in patients’ QoL (pooled RR = 1.60, 95% CI = 1.30–1.96) when compared to radiotherapy alone.

#### Various types of cancer

Four SRs reviewed the add-on effect of CHM on QoL among patients with different diagnoses. All SRs reported the add-on benefit of CHM on top of chemotherapy or radiotherapy in improving QoL, when compared to chemotherapy or radiotherapy alone. These SRs studied astragalus injection, Reishi mushroom extract, Xiaoaiping injection and SFI respectively.

#### Quality of evidence

Majority (93.5%) of the evidence reviewed is of moderate quality. Evidence on gastric cancer is of low quality, and in another SR on various types of cancer the evidence is of high quality.

### Survival rate

#### NSCLC

Four SRs reviewed various types of CHM plus chemotherapy versus chemotherapy alone for improving survival rate among NSCLC patients. All SRs showed that additional CHM treatment can slightly improve 1-year survival rate, although one SR reported that the benefit were not of significance. Other pooled results also showed that, CHM plus chemotherapy can significantly improve 2-year (pooled OR = 2.26, 95% CI = 1.16–3.99), 3-year (pooled OR = 2.59, 95% CI =  1.51–4.45) and 5-year (pooled OR = 2.45, 95% CI = 1.24–4.84) survival rates when compared to chemotherapy alone.

#### Lung cancer

One SR summarized evidence of various types of CHM plus chemotherapy or/and radiotherapy versus chemotherapy or/and radiotherapy alone for improving survival rate in lung cancer patients. Meta-analysis showed that CHM provide an add-on benefit in improving 2-year survival rate (pooled OR = 3.44, 95% CI = 2.04–5.80).

#### Liver cancer

Five SRs reviewed evidence of CHM plus TACE versus TACE alone for improving survival rate. CHM interventions included KS and mixed types of CHM. Meta-analyses showed that CHM provided additional benefits in improving 0.5-year, 1-year, 1.5-year, 2-year and 3-year survival rates. It should be noted that significant heterogeneities were found in three meta-analyses: 1.5-year survival rate (I^2^ = 70%, p = 0.009) in Wu 2009 b, 1.5-year survival rate (I^2^ = 63%, p = 0.03) and 3-year survival rate (I^2^ = 67%, p < 0.001) in Cheung 2013.

#### Gastric cancer

One SR reported that Kanglaite injection plus chemotherapy provide significant improvement on 1-year survival rate of gastric cancer patients (pooled OR = 6.74, 95 CI%  = 2.74–16.62) when compared to chemotherapy alone. Another SR found no significant benefit on 1-year survival rate (pooled RR = 1.25, 95% CI = 0.73–2.14) among TNM stage III–IV gastric patients using Huangchansu. Three other SRs included various types of CHM and meta-analyses showed that CHM plus chemotherapy can significantly improve 1-year, 2-year, 3-year and 5-year survival rates in gastric cancer patients.

#### Colorectal cancer

Four SRs summarized evidence on the add-on effect of CHM for improving survival rate. Meta-analyses showed that CHM plus chemotherapy provide significant greater improvement on the 0.5-year, 1-year, 2-year, 3-year, 4-year and 5-year survival rates, as compared to chemotherapy alone.

#### Nasopharyngeal cancer

Meta-analysis from a SR showed that, when compared to radiotherapy alone, combination of CHM and radiotherapy can slightly improve 3-year survival rate (pooled RR = 1.30, 95% CI = 1.03–1.63).

#### Esophageal cancer

One SR summarized evidence on KS. Although the addition of KS slightly improved 3-year survival rate (pooled OR = 1.86, 95% CI = 0.96–3.62) when used on top of chemotherapy, no significant difference was found when compared to chemotherapy alone.

#### Cervical cancer

A SR reported that, when compared to radiotherapy or chemotherapy alone, additional CHM treatment improved patients’ 1-year survival rate significantly (pooled OR = 4.16, 95% CI = 1.97–8.78).

#### Various types of cancer

One SR reviewed evidence on the add-on effect of KS among patients with various types of cancer. The results showed that KS only provide a small add-on improvement for 1-year and 2-year survival rates.

#### Quality of evidence

Only 28.9% of the evidence on prolonging survival time showed high quality, while 60.0% is of moderate quality and the remaining 11.1% is of low quality.

### Toxicities related to Chemotherapy or Radiotherapy

#### Leucopenia

##### NSCLC

Four SRs summarized evidence of the add-on effect of Aidi injection, SFI, Kanglaite injection and various types of CHM for reducing chemotherapy induced leucopenia (CIL). Meta-analyses demonstrated positive effect of these CHM in reducing CIL, but results from Kanglaite injection appeared to be heterogeneous (I^2^ = 52%, p = 0.03). Subgroup analysis from Li 2013 showed that CHM were more effective when the baseline severity of CIL is higher (pooled RR = 0.36 in CIL grade III–IV versus pooled RR = 0.75 in CIL grade I–IV).

##### Colorectal cancer

Results from a SR showed that CHM tends to be more effective in treating more severe CIL, but this trend is not reflected in a subgroup analysis including only patients with grade IV CIL. Another SR showed that, when compared to FOLFOX4 (5-Fluorouracil + Leucovorin + Oxaliplatin) alone, the additional use of CHM provides significant improvement on patients with grade III–IV neutropenia.

##### Gastric cancer

One SR showed significant add-on benefits of CHM in reducing grade II–IV CIL (pooled OR = 0.26, 95% CI = 0.18–0.37).

##### Various types of cancer

A SR summarized evidence on astragalus injection plus chemotherapy versus chemotherapy alone in reducing grade I–IV CIL for various types of cancer patients. Meta-analysis showed a slight add-on benefit from astragalus injection (pooled RR = 0.84, 95% CI = 0.79–0.88). Another SR reported that, when compared to chemotherapy alone, the addition of CHM significantly improved grade I–IV CIL (pooled OR = 0.40, 95% CI = 0.23–0.68). However, both meta-analyses had significant heterogeneity.

#### Nausea and vomiting

##### NSCLC

Evidence on CHM for reducing chemotherapy induced nausea and vomiting (CINV) were reviewed on Aidi injection, SFI, Kanglaite injection and various types of CHM. All meta-analyses showed favorable effect.

##### Liver cancer

Two SRs reviewed evidence of CHM for treating CINV. Both showed that CHM has a slightly positive effect in reducing CINV.

##### Colorectal cancer

Meta-analysis from a SR showed that when compared to chemotherapy alone, combination of CHM and chemotherapy significantly reduced grade III–IV CINV. Another SR reported that CHM tended to be more effective in managing more severe CINV, although no significant difference was seen in the outcomes of patients with grade I, II or IV CINV.

##### Gastric cancer

Evidence from one SR showed that the additional use of CHM provide a protective effect against grade II–IV CINV (pooled OR = 0.48, 95% CI = 0.34–0.66).

#### Thrombocytopenia

##### NSCLC

Two SRs reported that SFI and various types of CHM can significantly improve thrombocytopenia in NSCLC patients.

##### colorectal cancer

A SR included one RCT (n = 42) found that, when compared to FOLFOX4 alone, additional use of CHM has no effect in reducing grade III–IV thrombocytopenia (RR = 1.00, 95% CI = 0.07–14.95).

##### Gastric cancer

Evidence from a SR showed favorable effects of CHM in reducing grade II–IV thrombocytopenia (pooled OR = 0.35, 95% CI = 0.14–0.86).

##### Various types of cancer

Evidence showed the adjuvant use of astragalus injection or CHM can significantly reduce thrombocytopenia in cancer patients. Significant heterogeneity was seen in the first meta-analysis.

#### Anemia

##### NSCLC

Two SRs summarized evidence on SFI and CHM in preventing anemia. Results showed that the additional use of SFI significantly prevented the occurrence of grade III–IV anemia. However, this result is not consistent with results from another SR that summarized effects of a wide range of CHM. It is reported that CHM may significantly reduce grade I–IV anemia, but no significant difference was seen in the subgroup that only included grade III–IV patients.

##### Colorectal cancer

A SR showed that, when compared to FOLFOX4 alone, the additional use of CHM reduced the occurrence of grade III–IV anemia. However, no statistical difference was reached between the two groups.

##### Gastric cancer

Evidence from a SR showed that CHM plus chemotherapy can significantly reduce the occurrence of grade II–IV anemia, when compared to chemotherapy alone.

##### Various types of cancer

A SR suggested that the additional use of astragalus injection showed significant positive effect in reducing anemia grade I–IV in various types of cancer patients.

#### Neurotoxicity

##### Colorectal cancer

Although evidence showed that the additional use of CHM may reduce neurotoxicity of chemotherapy, no significant difference was reached in either of the two identified SRs.

#### Other chemotherapy related toxicity

##### Colorectal cancer

A SR summarized evidence on the combined use of CHM and FOLFOX4 for treating chemotherapy induced diarrhea and stomatitis in colorectal cancer patients. Although the additional use of CHM may reduce grade III–IV diarrhea and grade III–IV stomatitis, no statistical significant difference was reached in either meta-analysis.

##### Quality of evidence

More than half (65.9%) of evidence on CHM in reducing chemotherapy induced toxicity is of moderate quality, while 32.8% showed low quality and the remaining 2.3% is of very low quality.

#### Adverse effect of CHM

Among the 51 included SRs, seven (13.7%) described adverse effect from CHM usage. Three reported that no AE were described among the included RCTs. Four SRs reported a wide range of adverse effect, including nausea, insomnia, stomatitis, hair loss, mild gastric bleeding, low-grade fever, dizziness, gastrointestinal discomfort, mild skin itch and rashes. All these symptoms disappeared after discontinuing the CHM treatment, or alleviated after symptomatic treatment.

## Discussion

This overview summarized evidence on the effect of CHM for cancer palliative care, on top of conventional treatment. We identified SRs on nine types of cancer, of which evidence on 13 specific CHM intervention were included. We also described results from SRs that did not set any restrictions on cancer diagnoses nor types of CHM interventions, which may increase the external validity of this overview as this indicates the real world practice of traditional Chinese medicine. In general, results from the identified SRs demonstrated add-on benefit of CHM in improving QoL among patients with various types of cancer, including NSCLC, liver cancer, gastric cancer, colorectal cancer, breast cancer and nasopharyngeal cancer.

For survival, it is observed that the additional use of CHM significantly improved 2-year, 3-year and 5-year survival rates in NSCLC patients, 2-year survival rate in lung cancer patients, 0.5-year, 1-year, 1.5-year, 2-year and 3-year survival rates in liver cancer patients, 2-year, 3-year, 5-year survival rates in gastric cancer patients, 0.5-year, 1-year, 2-year, 3-year, 4-year and 5-year survival rates in colorectal cancer patients, 3-year survival rate in nasopharyngeal cancer patients, 1-year survival rate in cervical cancer patients. In SRs synthesizing evidence on various types of cancer, improvement on 1-year and 2-year survival rate were also observed. However, conflicting results were observed for the 1-year survival rates of NSCLC and gastric cancer patients. Also, there seems to be no add-on effect from KS in improving 3-year survival rate of patients with esophageal cancer.

Evidence showed that the combination of CHM and chemotherapy significantly reduced leucopenia, nausea and vomiting, thrombocytopenia and anemia in NSCLC, gastric cancer patients. It also significantly reduced nausea and vomiting in liver cancer patients. In general, CHM appears to be useful in improving leucopenia, thrombocytopenia and anemia among various types of cancer. Nevertheless, available evidence cannot demonstrate clear add-on benefits of CHM in improving leucopenia, nausea and vomiting, thrombocytopenia, anemia, neurotoxicity, diarrhea and stomatitis in colorectal cancer patients.

Base on the evidence we identified, CHM may be considered as an adjuvant option to improve QoL among cancer patients. Evidence showed inconsistency in the effectiveness of CHM for improving survival rate and reducing chemotherapy and/or radiotherapy related toxicity in cancer patients. Although we attempted to include all key outcomes on cancer palliative care, only three outcomes were identified in this overview. For some common symptoms which conventional care has limited options, such as pain, fatigue, anorexia, insomnia, limbs edema and constipation^8^,^14^, no relevant SR has been conducted. Finally, as the majority results were coming from Chinese population, the generalizability of the present results may be limited.

The methodological quality of included SRs was mediocre when compared to other SRs on CHM^15^ or those focused on conventional medicine^16^. Good performance was noted on conducting comprehensive literature search, and on assessing and documenting risk of bias of included studies, with more than 80% SRs satisfying these two criteria. On the other hand, improvement should be made in the remaining nine AMSTAR items, especially in providing a protocol, reporting lists of both included and excluded studies, and disclosing conflict of interests for both the SR and included studies. That said, quality of evidence is not as poor as we expected. Majority of evidence on improving QoL, prolonging survival time and reducing chemotherapy inducted toxicity are judged to be of moderate quality in terms of effectiveness.

In addition, reporting quality of included SRs was unsatisfactory, often with little details on CHM and conventional treatments provided, as well as on how outcomes were measured. Future SR should comply with the PRISMA statement^17^,^18^ such that it is more useable for policy makers and clinicians. Another limitation of the included SRs is that the majority of them did not mention results on CHM safety, only seven (13.7%) SRs reported adverse effects which were originated from CHM usage. Results from these seven SRs indicated that adverse events from CHM were mild, but a firm conclusion on the safety of CHM usage cannot be made as discovery of rare and longer term toxicities would require case-control and retrospective cohort designs. In the future, well reported observational studies and RCTs are needed to clarify the presence of short and long term toxicities of CHM.

Limitations on reporting were also noted among trials included in the SRs. Many trials were judged to have unclear risk of bias by the systematic reviewers, reflecting the lack of compliance to the CONSORT guideline for reporting^19^,^20^,^21^,^22^. It is unfortunate that poor reporting practice does not improve despite the availability of Chinese CONSORT^23^. This has limited us from excluding SRs that mainly report results from trials with high risk of bias, and the potential impact of this on the trustworthiness of the results should be highlighted. In addition, since the clinical evidence presented in all SRs were mainly obtained from trial reports published in mainland China, there was a potential risk of positive publication bias^22^,^24^, although this phenomenon is not restricted to Chinese publications^25^. To prevent publication bias, it is recommended that all clinical trials protocols on the topic should register with a recognized platform (e.g. the Chinese Clinical Trial Registry)^26^.

Although we did not set restriction on QoL measurement tool in our eligibility criteria, all included SRs reported such outcome using the KPS. KPS only measure general performance status, which might not be sufficient for assessing QoL of cancer patients comprehensively. Future trials are suggested to adopt more specific QoL measurement tool such as the Short Form 36 questionnaire, and the European Organisation for Research and Treatment of Cancer Quality of Life Questionnaire-core 30^27^.

Major contributions of this overview are to comprehensively summarize; and to critically appraise all available evidence on CHM for cancer palliative care. Limitations on both reporting and methodological rigor of the existing SRs as well as primary studies were identified and suggestions were made on how these can be improved of future studies on this area. Clinical questions that are waiting for further researches were also identified through this study.

In conclusion, current clinical evidence indicated that CHM may be considered as a palliative care option for improving QoL among cancer patients. There are conflicting results on the effectiveness of CHM in prolonging survival and in reducing chemotherapy and/or radiotherapy related toxicities. Quality of evidence is moderate for these three outcomes. Also, future trials are suggested to investigate the effectiveness of CHM in managing common symptoms like pain, fatigue, anorexia, insomnia, limbs edema and constipation^8^,^14^, in which conventional care options for these common cancer related symptoms were limited. Methodological quality of SRs in CHM for cancer palliative care is not satisfactory. To provide more rigorous evidence on the effectiveness of CHM, future SRs and trials must adhere to high methodological and reporting standards.

## Methods

### Criteria for considering meta-analyses for inclusion

This overview only included SR with meta-analysis that quantitatively summarized evidence on CHM for cancer palliative care. SR is defined as an “attempt to identify, appraise and synthesize all the empirical evidence that meets pre-specified eligibility criteria to answer a given research question”, in accordance to the Cochrane Handbook version 5.1.0.^28^ Any SRs that meet the following criteria were included in this overview: i) Cochrane SR or non-Cochrane SR focusing on cancer palliative care with meta-analysis conducted; ii) meta-analyses must pooled clinical trials that evaluate the effectiveness of at least one CHM indexed in the 2010 China Pharmacopeia Chinese herbal medicine index^29^. The protocol of this overview has been registered in PROSPERO (http://www.crd.york.ac.uk/PROSPERO/display_record.asp ?ID = CRD42015016171).

### Participants

SR including clinical trials on patients diagnosed with any type of cancer and received at least one form of CHM for supportive or palliative care was considered eligible.

### Interventions & control

CHM of any dosage form or route of administration was considered eligible in this overview. We included SRs that include studies providing any type of control treatment without CHM. These interventions include conventional treatment, placebo, chemotherapy, radiotherapy, or no treatment.

### Outcomes of interest

With an aim to provide a comprehensive picture of available clinical evidence on CHM for cancer palliative care, we included all cancer or treatment related outcomes measured using validated approaches. Special attention was paid to those symptoms that are frequently experienced by cancer patients but limited treatment choices are available from conventional medicine. These outcomes include cancer related pain, fatigue, anorexia, insomnia, limbs edema and constipation^8^,^14^.

### Literature search

We conducted a comprehensive literature search in seven databases from their inception till July 2014. Both international databases (MEDLINE, EMBASE, Cochrane Database of Systematic Reviews (CDSR) and Database of Abstracts of Reviews of Effect (DARE)) and Chinese databases (Chinese Biomedical Databases (CBM), Wan Fang Digital Journals and Taiwan Periodical Literature Databases) were searched to identify potential SRs. Specialized search filter for reviews were used for MEDLINE and EMBASE^30^. Detailed search strategies are reported in Appendix 1.

### Literature selection, data extraction, methodological quality and quality of evidence assessment

All retrieved citations were screened and assessed for their eligibility according to the inclusion criteria. For duplicate publications, the most updated version was selected.

The following data were extracted from each included SR: i) basic characteristics of the SR, including search date, number of included studies, total number of patients and bibliographic information; ii) detailed information on study design and patient, intervention, control and outcomes; and iii) statistical results, including pooled effects of each comparison for each outcome.

Methodological quality of included SRs was assessed with the Methodological Quality of Systematic Reviews (AMSTAR) Instrument^31^, which was shown to be a reliable and valid tool for assessing the methodological quality of SRs^32^,^33^. Eleven aspects were assessed by using AMSTAR, with each aspect being judged as yes, no, can’t answer or not applicable based on the information provided. Detailed operational guide for AMSTAR is provided in Appendix 2.

Quality of evidence for each outcome was assessed using the Chinese and Integrative Medicine Evidence Rating System (CHIMERAS)^34^. Quality of evidence was judged across five levels (very low, low, moderate, high and very high) by considering rigors of both qualitative (direction of effect) and quantitative (effect size) conclusions.

Literature selection, data extraction, methodological quality and quality of evidence assessment were conducted by two researchers independently, with any disagreement resolved by discussion and consensus. Unresolved discrepancy was managed by a third reviewer.

### Data synthesis

The effectiveness of CHM treatments was assessed at review level. We did not re-analyze the data of the primary trials included in the SR. We extracted the pooled effect estimation from each meta-analysis. Pooled relative risk (RR) or odds ratio (OR) for dichotomous outcomes, and mean difference (MD) for continuous outcomes accompanied with respective 95% confidence interval (CI) were reported.

## Additional Information

**How to cite this article**: Chung, V. C.H. *et al.* Effectiveness of Chinese herbal medicine for cancer palliative care: overview of systematic reviews with meta-analyses. *Sci. Rep.* **5**, 18111; doi: 10.1038/srep18111 (2015).

## Supplementary Material

Appendix 1-4

## Figures and Tables

**Table 1:** Characteristics of included meta-analyses on Chinese herbal medicine for cancer palliative care.

First author and year of publication	Included study design	Search period	Cancer site (tumor stage)	No. of studies (No. of patients)	Nature of Chinese herbal medicine (CHM) interventions^	Nature of control interventions	Outcomes reported
Ma, 2004	RCT or quasi-RCT	2003	NSCLC (TNM III–IV)	10 (771)	CHM + chemotherapy (Cisplatin drugs)	Cisplatin drugs chemotherapy alone	QOL
Liu,2009	RCT	Sep. 2007	CRC (NR)	6 (334)	Jianpi CHM + chemotherapy	Chemotherapy alone	Chemotherapy related toxicity, including: leucopenia, nausea and vomiting, neurotoxicity
Wu, 2009a	RCT	Jul. 2008	NSCLC (TNM III–IV)	19 (1380)	Aidi injection + NP chemotherapy	NP chemotherapy alone	chemotherapy related toxicity: leucopenia, nausea and vomiting
Wu, 2009b	RCT	Feb. 2009	Liver cancer (TNM III–IV)	45 (3236)	CHM + TACE	TACE alone	Survival rate
Zhu,2009	RCT or quasi-RCT	Aug. 2008	NSCLC (TNM III–IV)	16 (1041)	KLT+ NP or MVP or GP chemotherapy	NP or MVP or GP chemotherapy alone	QOL
Chen, 2010	RCT	Oct. 2009	NSCLC (NR)	11 (796)	CHM+ MVP or NP chemotherapy	MVP or NP chemotherapy alone	Survival rate
Dong, 2010a	RCT or quasi-RCT	NR	Various (NR)	12 (1230)	Astragalus injection + chemotherapy	Chemotherapy alone	QOL, chemotherapy related toxicity: leucopenia, anemia, thrombocytopenia
Fu, 2010	RCT	Oct. 2009	Various (NR)	24 (4150)	CHM + chemotherapy	Chemotherapy alone or Chemotherapy + placebo	Chemotherapy related toxicity: leucopenia, thrombocytopenia
Guo, 2010	RCT	2009	Liver cancer (NR)	33 (2619)	CHM + TACE	TACE alone or TACE + placebo	QOL
Zhou, 2010	RCT	NR	Gastric cancer (NR)	13 (877)	CHM + chemotherapy	Chemotherapy alone	Survival rate
Dong, 2010b	RCT	Apr. 2010	NSCLC (TNM III–IV)	29 (2062)	SFI + platinum containing chemotherapy	Platinum containing chemotherapy alone	QOL, chemotherapy related toxicity: leucopenia, anemia, thrombocytopenia, and nausea and vomiting
Cui, 2011	RCT	Dec. 2010	NSCLC (TNM III–IV)	9 (584)	KS + NP chemotherapy	NP chemotherapy alone	QOL
Ma, 2011a	RCT	Jun. 2010	NSCLC (TNM III–IV)	11 (752)	KS + NP chemotherapy	NP chemotherapy alone	QOL
Ma, 2011b	RCT	Jun. 2010	Liver cancer (NR)	11 (NR)	KS + TACE	TACE alone	QOL, survival rate
Wang, 2011	RCT	Aug. 2010	Gastric cancer (NR)	4 (304)	KLT + chemotherapy	Chemotherapy alone	Survival rate
Qiao, 2011	RCT	Mar. 2010	Nasopharyngeal cancer (TNM I–IV)	9 (795)	Yiqi Yangyin and Qingre Huoxue decoction + radiotherapy	Radiotherapy alone	QOL, survival rate
Zhuang, 2011	RCT or qusai-RCT	Nov. 2010	NSCLC (TNM III–IV)	6 (416)	Kang Ai injection + TP	TP chemotherapy alone	QOL
Guo, 2012	RCT or quasi-RCT	Aug. 2011	CRC (TNM IV)	20 (1304)	CHM + chemotherapy or radiotherapy	Chemotherapy or radiotherapy alone	Survival rate, QOL
Jin, 2012	RCT	Oct. 2011	Various (NR)	5 (373)	Extract of *Ganoderma lucidum* (chemotherapy or radiotherapy)	Chemotherapy + placebo or chemotherapy alone or radiotherapy+ conventional care	QOL
Yang, 2012	RCT or quasi-RCT	2006	Lung cancer (NR)	10 (853)	CHM* + chemotherapy or radiotherapy or both	Chemotherapy or radiotherapy or both.	Survival rate
Qin, 2012	RCT	Oct. 2011	NSCLC (TNM III–IV)	13 (972)	Kang Ai injection + paclitaxel or gemcitabine or navelbin + platinum containing chemotherapy	Paclitaxel or gemcitabine or navelbin + platinum containing chemotherapy alone	QOL
Cai, 2012	RCT	2011	Gastric cancer (TNM I–IV)	9 (NR)	SFI + chemotherapy	Chemotherapy alone	QOL
Wang, 2012a	RCT or qusai-RCT	2010	CRC (NR)	9 (518)	CHM + chemotherapy	Chemotherapy alone	QOL
Li, 2012a	RCT or quasi-RCT	2011	CRC (NR)	14 (1081)	CHM+ chemotherapy	Chemotherapy alone	Survival rate
Li, 2012b	RCT	Aug. 2011	Liver cancer (NR)	47 (3854)	CHM + TACE	TACE alone	Survival rate, chemotherapy related toxicity: nausea and vomiting
Liu, 2012	RCT	2011	Breast cancer (NR)	6 (NR)	KS + chemotherapy	Chemotherapy alone	QOL
Fan, 2012	RCT	Jun. 2011	Breast cancer (TNM I–IV)	6 (496)	KS + chemotherapy	Chemotherapy alone	QOL
Ma, 2012	RCT	Jun. 2010	NSCLC (TNM III–IV)	8 (696)	KS +TP chemotherapy	TP chemotherapy alone	QOL
Shi, 2012	RCT or quasi-RCT	2010	Gastric cancer (NR)	21 (1178)	CHM + chemotherapy	Chemotherapy alone	Survival rate, Chemotherapy related toxicity: leucopenia, anemia, thrombocytopenia.
Wang, 2012b	RCT	Jun. 2012	Gastric cancer (TNM III–IV)	44 (3088)	CHM + conventional care	Conventional care alone	QOL
Rong, 2012	RCT	Nov. 2011	NSCLC (TNM III–IV)	18 (1108)	CHM + Chemotherapy	Chemotherapy alone	Survival rate
Wang, 2012c	RCT or qusai-RCT	Sep. 2011	Various (NR)	11 (618)	Xiaoaiping injection + chemotherapy	Chemotherapy alone	QOL
Zhang, 2012	RCT	Dec. 2011	Various (TNM II–IV)	16 (1539)	SFI + chemotherapy or radiotherapy	Chemotherapy or radiotherapy alone	QOL
Xu, 2012	RCT or qusai-RCT	NR	Cervical cancer (TNM II–IV)	18 (1657)	CHM + radiotherapy or CHM + radiotherapy + conventional care	Radiotherapy alone or radiotherapy + conventional care	Survival rate
Cheung, 2013	RCT	Oct. 2012	Liver cancer (TNM II–IV)	67 (5211)	CHM + TACE	TACE alone	QOL, survival rate, Chemotherapy related toxicity: nausea and vomiting
Li, 2013	RCT	Sep. 2012	NSCLC (TNM III–IV)	24 (NR)	CHM + chemotherapy	Chemotherapy alone	QOL, survival rate, Chemotherapy related toxicity: nausea and vomiting, leucopenia, anemia and thrombocytopenia.
Xie, 2013	RCT or quasi-RCT	Jan. 2013	Gastric cancer (TNM III–IV)	15 (1008)	Huachansu + chemotherapy	Chemotherapy alone	Survival rate
Du, 2013	RCT	2012	Esophageal cancer (NR)	5 (NR)	KS + chemotherapy or radiotherapy	chemo or radiotherapy or (chemo + radiotherapy) alone	Survival rate
Tian, 2013	RCT	Nov. 2012	NSCLC (NR)	24 (1845)	CHM + chemotherapy or radiotherapy	chemotherapy alone or chemotherapy + radiotherapy	Survival rate
Jiang, 2013	RCT	2013	Liver cancer (NR)	16 (1105)	CHM + TACE	TACE alone	Survival rate
Liu, 2013	RCT or quasi-RCT	2012	NSCLC (NR)	6 (346)	Zilongjin (bailongpian) + chemotherapy	chemotherapy (GP, NP or MVP, MVP) alone	QOL
Xu, 2013	RCT	Oct. 2012	Gastric cancer (NR)	15 (NR)	CHM + Chemotherapy	Chemotherapy alone	Survival rate
Xiao, 2013	RCT	2012	NSCLC (NR)	10 (588)	Xiaoaiping injection + platinum containing chemotherapy	Platinum containing chemotherapy alone	QOL
Su, 2013	RCT or quasi-RCT	Oct. 2012	Various (NR)	82 (NR)	KS + chemotherapy or radiotherapy	Chemotherapy or radiotherapy alone	Survival rate
Yan, 2013	RCT	Feb. 2012	NSCLC (TNM III–IV)	10 (687)	KLT+NP chemotherapy	NP chemotherapy alone	QOL
Sheng, 2013	RCT	2011	NSCLC (NR)	38 (NR)	SFI + chemotherapy (GP or NP or TP or DP)	Chemotherapy (GP or NP or TP or DP) alone	QOL
He, 2013	RCT	Sep. 2012	NSCLC (TNM III–IV)	19 (1110)	Shenfu injection + platinum containing chemotherapy	Platinum containing chemotherapy alone	QOL
Chen, 2014	RCT	Dec. 2012	CRC (TNM IV)	13 (940)	CHM + FOLFOX 4	FOLFOX4 alone	Survival rate, Chemotherapy related toxicity: neutropenia, nausea and vomiting, neurotoxicity, diarrhea, anemia, thrombocytopenia, and stomatitis
Xu, 2014	RCT	Dec. 2012	NSCLC (TNM III–IV)	17 (1605)	KLT + chemotherapy	Chemotherapy alone	Chemotherapy related toxicity: nausea and vomiting, leucopenia
Liu, 2014	RCT	Jun. 2012	CRC (TNM IV)	13 (781)	CHM + chemotherapy	Chemotherapy alone	Survival rate
Wang, 2014	RCT	2012	Gastric cancer (TNM III–IV)	10 (1020)	SFI+FOLFOX4	FOLFOX4 alone	QOL

^*^Included all types of CHM.

^^^CHM denotes the inclusion of all types of Chinese herbal medicines. The use of generic terms “chemotherapy” or “radiotherapy” denotes that the specific treatments used were not described in the original meta-analyses publications.

Keys: CHM, Chinese herbal medicine; CRC, colorectal cancer; DP, Docetaxel + Cisplatin; FOLFOX, the FOLFOX regimen refers to 5-Fluorouracil + Leucovorin combined with Oxaliplatin; GP, Gemcitabine + Cisplatin; KLT, Kanglaite injection; KS, Compound Kushen injection; MVP, Mitomycin + Vindesine + Cisplatin; NP, Cisplatin + Vinorelbine; NR, not reported; NSCLC, non-small lung cell cancer; QOL, quality of life; RCT, randomized controlled trial; SFI, Shenqi Fuzheng injection; SR, systematic review; TACE, Transcatheter arterial chemoembolization; TNM, tumor-node-metastasis stage; TP, Paclitaxel + Cisplatin.

**Table 2:** Methodological quality of included meta-analyses on Chinese herbal medicine for cancer palliative care.

First author and year of publication	AMSTAR item										
	1	2	3	4	5	6	7	8	9	10	11
Ma, 2004	N	NR	Y	NR	N	N	N	N	Y	N	N
Liu, 2009	N	Y	Y	N	N	N	N	Y	Y	N	N
Wu, 2009a	N	NR	NR	NR	N	N	Y	Y	NR	Y	N
Wu, 2009b	N	Y	Y	Y	N	N	NR	Y	NR	Y	N
Zhu, 2009	N	NR	Y	Y	N	N	Y	Y	Y	Y	N
Chen, 2010	N	NR	N	NR	N	N	N	N	N	Y	N
Dong, 2010	N	Y	Y	Y	N	N	Y	Y	N	Y	N
Fu, 2010	N	Y	Y	N	N	N	Y	N	Y	Y	N
Guo, 2010	N	NR	Y	NR	N	N	Y	Y	N	Y	N
Zhou, 2010	N	NR	Y	N	N	N	Y	N	Y	N	N
Dong, 2010	N	Y	Y	N	N	N	Y	Y	Y	Y	N
Cui, 2011	N	Y	Y	Y	N	N	Y	Y	Y	Y	N
Ma, 2011a	N	NR	Y	NR	N	N	Y	Y	Y	Y	N
Ma, 2011b	N	NR	Y	NR	N	N	Y	Y	Y	Y	N
Wang, 2011	N	Y	Y	NR	N	Y	Y	N	Y	N	N
Qiao, 2011	N	Y	Y	Y	N	N	Y	Y	Y	Y	N
Zhuang, 2011	N	Y	Y	NR	N	N	Y	Y	Y	Y	N
Qin, 2012a	Y	Y	Y	Y	Y	Y	Y	N	Y	N	N
Jin, 2012	Y	Y	Y	Y	Y	N	Y	Y	Y	N	N
Yang, 2012	N	NR	Y	N	N	N	Y	N	Y	N	N
Qin, 2012b	N	NR	Y	N	N	N	Y	N	Y	Y	N
Cai, 2012	N	Y	Y	Y	N	Y	Y	Y	Y	N	N
Wang, 2012a	N	NR	Y	N	N	N	Y	N	Y	Y	N
Li, 2012a	N	Y	Y	N	N	Y	Y	Y	Y	Y	N
Li, 2012b	N	Y	Y	Y	N	Y	Y	Y	Y	N	N
Liu, 2012	N	NR	Y	N	N	N	Y	Y	Y	Y	N
Fan, 2012	N	NR	Y	Y	N	Y	Y	N	Y	N	N
Ma, 2012	N	NR	Y	NR	N	N	Y	Y	Y	Y	N
Shi, 2012	N	Y	Y	N	N	N	Y	Y	N	N	N
Wang, 2012b	N	Y	Y	N	N	N	NR	N	Y	Y	N
Rong, 2012	N	Y	Y	N	N	N	Y	N	Y	Y	N
Wang, 2012c	N	NR	Y	N	N	N	Y	N	Y	Y	N
Zhang, 2012	N	Y	Y	N	N	Y	Y	Y	Y	Y	N
Xu, 2012	N	Y	Y	Y	N	N	Y	Y	Y	N	N
Cheung, 2013	N	Y	Y	Y	N	Y	Y	Y	N	Y	N
Li, 2013	N	Y	Y	NR	N	Y	Y	Y	Y	N	N
Xie, 2013	N	Y	Y	Y	N	Y	NR	N	NR	Y	N
Du, 2013	N	Y	Y	NR	N	N	Y	Y	N	Y	N
Tian, 2013	N	Y	Y	Y	N	N	Y	Y	Y	Y	N
Jiang, 2013	N	NR	Y	N	N	N	Y	N	Y	Y	N
Liu, 2013	N	Y	Y	Y	N	N	Y	Y	N	N	N
Xu, 2013	N	NR	Y	N	N	N	Y	N	Y	N	N
Xiao, 2013	N	Y	Y	Y	N	Y	Y	Y	Y	N	N
Su, 2013	N	Y	Y	NR	N	Y	Y	Y	Y	Y	N
Yan, 2013	N	NR	Y	NR	N	N	Y	N	Y	Y	N
Sheng, 2013	N	Y	Y	Y	N	N	Y	Y	Y	Y	N
He, 2013	N	Y	Y	Y	N	Y	Y	Y	Y	N	N
Chen, 2014	N	Y	Y	Y	N	Y	Y	Y	NR	Y	N
Xu, 2014	N	Y	Y	N	N	Y	Y	N	N	Y	N
Liu, 2014	N	NR	Y	NR	N	N	NR	N	Y	N	N
Wang, 2014	N	Y	Y	N	N	Y	Y	Y	Y	Y	N
# of Yes (%)	2 (3.9)	32 (62.7)	49 (96.1)	19 (37.3)	2 (3.9)	16 (31.4)	44 (86.3)	31 (60.8)	39 (76.5)	33 (64.7)	0 (0.0)

Keys: CHM, Chinese herbal medicine; N, no; NR, not reported; Y, yes (meta-analysis fulfilling the criteria); # of Yes, number of yes; AMSTAR item: 1. Was an ‘a priori’ design provided? 2. Was there duplicate study selection and data extraction? 3. Was a comprehensive literature search performed? 4. Was the status of publication (i.e. grey literature) used as an inclusion criterion? 5. Was a list of studies (included and excluded) provided? 6. Were the characteristics of the included studies provided? 7. Was the scientific quality of the included studies assessed and documented? 8. Was the scientific quality of the included studies used appropriately in formulating conclusions? 9. Were the methods used to combine the findings of studies appropriate? 10. Was the likelihood of publication bias assessed? 11. Was the conflict of interest included?

**Table 3:** Chinese Herbal Medicine for Improving QOL among Cancer Patients: Overview of Meta-Analyses Results.

First author and year of publication	Comparison^	Outcome assessment method	No. of studies (No. of patients)	Pooled results (95% CI)	Heterogeneity I^2^ (%)	Quality of evidence
*Non-small lung cell cancer*						
Cui, 2011	KS+NP vs. NP	Improvement rate*	5(NR)	OR: 2.38 [1.43, 3.95]	0.0	Moderate
Ma, 2011a	KS+NP vs. NP	Improvement rate	7(537)	OR: 2.78 [1.87, 4.15]	0.0	Moderate
Ma, 2012b	KS+TP vs. TP	Improvement rate	6(475)	OR: 3.26 [2.22, 4.80]	0.0	Moderate
Zhu, 2009	KLT+NP vs. NP	Responder rate #	4(234)	RR: 1.34 [1.14, 1.58]	0.0	Moderate
Yan, 2013	KLT+NP vs. NP	Improvement rate	7(505)	RR: 1.73 [1.34, 2.23]	57.0¶	Moderate
Dong, 2010b	SFI + platinum containing chemotherapy vs. platinum containing chemotherapy	Responder rate	20(1336)	RR: 1.57 [1.45, 1.70]	24.8	Moderate
Sheng, 2013	SFI + chemotherapy vs. chemotherapy	Responder rate	27(1805)	RR: 1.40 [1.30, 1.52]	44.0	Moderate
Zhuang, 2011	Kang Ai injection +TP vs. TP	Improvement rate	5(356)	OR: 3.13 [1.88, 5.20]	0.0	Moderate
Qin, 2012b	Kang Ai injection+ chemotherapy vs. chemotherapy	Improvement rate	11(804)	OR: 1.87 [1.60, 2.19]	0.0	Moderate
Ma, 2004	CHM + chemotherapy vs. chemotherapy	Responder rate	7(555)	OR: 3.36 [2.47, 4.57]	NR	Moderate
Li, 2013	CHM+ chemotherapy vs. chemotherapy	Improvement rate	6(526)	RR: 3.25 [2.22, 4.77]	51.0	Moderate
Liu, 2013	Zijinglong + chemotherapy vs. chemotherapy	Improvement rate	6(346)	RR: 4.14 [2.80, 6.12]	77.0¶	Moderate
	Zijinglong + MVP vs. MVP	Improvement rate	3(150)	RR: 12.72[4.70, 34.43]	42.0	Moderate
Xiao, 2013	Xiaoaiping injection+platinum containing chemotherapy vs. platinum containing chemotherapy	Improvement rate	10(588)	OR: 1.57 [1.12, 2.20]	0.0	Moderate
He, 2013	Shenfu Injection +chemotherapy vs. chemotherapy	Improvement rate	3(198)	OR: 2.72 [1.48, 5.00]	0.0	Moderate
*Liver cancer*						
Ma, 2011b	KS+TACE vs. TACE	Improvement rate	6(447)	OR: 2.58 [1.71, 3.89]	0.0	Moderate
Guo,2010	CHM+TACE vs. TACE	Improvement rate	26(1882)	OR: 1.78 [1.58, 2.01]	0.0	Moderate
Cheung, 2013	CHM+TACE vs. TACE	Improvement rate	27(2014)	RR: 1.74 [1.57, 1.93]	0.0	Moderate
		KPS score	9(477)	MD: 10.03 [8.98, 11.07]	95.0¶	Moderate
*Gastric cancer*						
Cai, 2012	SFI+ chemotherapy vs. chemotherapy	Improvement rate	8(534)	RR: 3.14 [2.11, 4.69]	7.0	Moderate
Wang, 2014	SFI+FLOFOX4 vs. FLOFOX4	Responder rate	6(505)	OR: 1.48 [1.26, 1.57]	33.0	Moderate
Wang, 2012b	CHM+ conventional care vs. conventional care	KPS score	17(1359)	MD: 0.51 [0.21, 1.82]	86.0¶	Low
*Colorectal cancer*						
Guo, 2012	CHM + chemotherapy vs. chemotherapy	Improvement rate	8(605)	RR: 1.85 [1.55, 2.21]	NR	Moderate
Wang, 2012a	CHM+ chemotherapy vs. chemotherapy	Improvement rate	6(282)	OR: 3.48 [2.17, 5.58]	0.0	Moderate
*Breast cancer*						
Liu, 2012	KS+ chemotherapy vs. chemotherapy	Improvement rate	4(370)	OR: 2.98 [1.85,4.80]	0.0	Moderate
Fan, 2012	KS+ chemotherapy vs. chemotherapy	Improvement rate	4 (316)	RR: 3.44 [2.09, 5.67]	0.0	Moderate
*Nasopharyngeal cancer*						
Qiao, 2011	CHM + radiotherapy vs. radiotherapy	Responder rate	2(166)	RR: 1.60 [1.30, 1.96]	0.0	Moderate
*Various types of cancer*						
Dong, 2010a	Astragalus extract + chemotherapy vs. chemotherapy	Responder rate	6(641)	RR: 1.46 [1.29, 1.66]	39.1	Moderate
Jin, 2012	*Ganoderma lucidum* extract + chemotherapy or radiotherapy vs. chemotherapy or radiotherapy	Improvement rate	3(284)	RR: 2.51 [1.86, 3.40]	48.0.	High
Wang, 2012c	Xiaoaiping injection+ chemotherapy vs. chemotherapy	Improvement rate	9(568)	RR: 1.80 [1.49, 2.18]	27.0	Moderate
Zhang, 2012	SFI+ chemotherapy or radiotherapy vs. chemotherapy or radiotherapy	Improvement rate	7(623)	OR: 3.07 [2.15, 4.39]	0.0	Moderate

^*^QOL measured with the Karnofsky Performance Status (KPS) scale. A KPS score increment >10 is defined as an improvement. Improvement rate  =  number of patients who had a KPS increment >10 / total number of patients, this definition apply to all improvement rate in Table 3.

^#^A KPS score increment >0 is defined as an improvement. Responder rate  =  number of patients who had a KPS increment >0 / total number of patients, this definition apply to all improvement rate in Table 3.

^¶^p < 0.05 for the heterogeneity test;

^^^CHM denotes the inclusion of all types of Chinese herbal medicines. The use of generic terms “chemotherapy” or “radiotherapy” denote that the specific treatments used were not described in the original meta-analyses publications.

Keys: CHM, Chinese herbal medicine; CI confidence interval; FOLFOX, the FOLFOX regimen refers to 5-Fluorouracil + Leucovorin combined with Oxaliplatin; KLT, Kanglaite injection; KS, Compound Kushen injection; MD, mean difference; MVP, Mitomycin + Vindesine + Cisplatin; NP, Cisplatin + Vinorelbine; NR, not reported; OR, odds ratio; QOL, quality of life; RR, relative risk; SFI, Shenqi Fuzheng injection; TACE, Transcatheter arterial chemoembolization; TP, Paclitaxel + Cisplatin.

**Table 4 t4:** Chinese Herbal Medicine for Improving Survival Rate among Cancer Patients: Overview of Meta-Analyses Results.

First author and year of publication	Comparison^	Duration of follow up (year)	No. of studies (No. of patients)	Pooled results (95% CI)	Heterogeneity I2 (%)	Quality of evidence
*Non-small cell lung cancer*						
Chen, 2010	CHM+ chemotherapy vs. chemotherapy	1	4(338)	OR: 1.29 [0.83, 2.01]	0.0	Low
CHM+ chemotherapy vs. chemotherapy	2	2(180)	OR: 2.26 [1.16, 3.99]	58.	Low	
Rong, 2012	CHM+ chemotherapy vs. chemotherapy	1	5(NR)	RR: 1.35[1.09, 1.66]	0.0	Moderate
Li, 2013	CHM+ chemotherapy vs. chemotherapy	1	7(608)	RR: 1.36[1.15, 1.60]	0.0	Moderate
Tian, 2013	CHM+ chemotherapy vs. chemotherapy	1	5(NR)	OR: 1.56[1.08, 2.25]	0.0	Moderate
	CHM+ chemotherapy vs. chemotherapy	3	5(NR)	OR: 2.59[1.51, 4.45]	0.0	High
	CHM+ chemotherapy vs. chemotherapy	5	5(NR)	OR: 2.45[1.24, 4.84]	0.0	High
*Lung cancer (Type unspecified)*						
Yang, 2012	CHM+ chemotherapy vs. chemotherapy	2	4(406)	OR: 3.44[2.04, 5.80]	0.0	Moderate
*Liver cancer*						
Wu, 2009b	CHM+TACE vs. TACE	0.5	15(NR)	RR: 1.10[1.04, 1.15]	0.0	Moderate
	CHM+TACE vs. TACE	1	22(NR)	RR: 1.26[1.17, 1.36]	7.0	Moderate
	CHM+TACE vs. TACE	1.5	4(NR)	RR: 1.71[1.02, 2.91]	70.0*	Low
	CHM+TACE vs. TACE	2	15(NR)	RR: 1.72[1.40, 2.03]	0.0	Moderate
	CHM+TACE vs. TACE	3	8(NR)	RR: 2.40[1.65, 3.49]	0.0	High
Ma, 2011b	KS+TACE VS. TACE	1	4(283)	OR: 2.18 [1.29, 3.69]	0.0	High
Li, 2012b	CHM+TACE vs. TACE alone or TACE+ conventional care	1	17(1238)	RR: 1.36[1.25, 1.49]	15.0	Moderate
*Liver cancer*						
Cheung, 2013	CHM+TACE vs. TACE	0.5	22(2278)	RR: 1.12 [1.07, 1.16]	0.0	Moderate
	CHM+TACE vs. TACE	1	30(2963)	RR: 1.40 [1.32, 1.50]	0.0	Moderate
	CHM+TACE vs. TACE	1.5	5(327)	RR: 1.89 [1.44, 2.49]	63.0*	Moderate
	CHM+TACE vs. TACE	2	19(2220)	RR: 1.75 [1.55, 1.99]	30.0	Moderate
	CHM+TACE vs. TACE	3	11(1338)	RR: 2.51 [1.97, 3.19]	67.0*	High
Jiang, 2013	CHM+TACE vs. TACE	1	12(991)	OR: 2.15[1.63, 2.85]	21.0	High
*Gastric cancer*						
Zhou,2010	CHM+ chemotherapy vs. chemotherapy	3	4(409)	OR: 2.33 [1.53, 3.56]	22.4	Moderate
	CHM+ chemotherapy vs. chemotherapy	5	5(655)	OR: 1.84 [1.31, 2.59]	0.0	Moderate
Wang, 2011	KLT + chemotherapy vs. chemotherapy	1	2(94)	OR: 6.74 [2.74, 16.62]	0.0	High
Shi, 2012	CHM+ chemotherapy vs. chemotherapy	1	3(289)	OR: 1.10[1.01, 1.21]	0.0	Low
	CHM+ chemotherapy vs. chemotherapy	2	3(289)	OR: 1.29[1.11, 1.50]	0.0	Moderate
	CHM+ chemotherapy vs. chemotherapy	3	3(311)	OR: 1.43[1.15, 1.75]	0.0	Moderate
Xie, 2013	Huachansu + chemotherapy vs. chemotherapy	1	4(NR)	RR: 1.25[0.73, 2.14]	0.0	Moderate
Xu, 2013	CHM+ chemotherapy vs. chemotherapy	1	4(399)	OR: 2.17[1.15, 4.08]	0.0	Moderate
	CHM+ chemotherapy vs. chemotherapy	3	4(407)	OR: 2.26[1.51, 3.39]	0.0	Moderate
*Colorectal cancer*						
Guo, 2012	CHM + chemotherapy vs. chemotherapy	1	4 (238)	RR: 1.39[1.15, 1.69]	NR	Moderate
	CHM + chemotherapy vs. chemotherapy	3	2(129)	RR: 2.23 [1.05, 4.73]	NR	Moderate
Li, 2012a	CHM+ chemotherapy vs. chemotherapy	0.5	3(134)	OR: 2.19[1.10, 4.34]	0.0	High
	CHM+ chemotherapy vs. chemotherapy	1	12(930)	OR: 2.83[2.01, 3.99]	0.0	High
	CHM+ chemotherapy vs. chemotherapy	2	6(454)	OR: 2.59[1.59, 4.24]	0.0	High
	CHM+ chemotherapy vs. chemotherapy	3	8(657)	OR: 2.25[1.54, 3.30]	0.0	High
	CHM+ chemotherapy vs. chemotherapy	4	1(122)	OR: 2.29[1.08, 4.82]	NA	High
	CHM+ chemotherapy vs. chemotherapy	5	4(394)	OR: 2.32[1.55, 3.48]	0.0	High
Chen, 2014	CHM+FOLFOX4 vs. FOLFOX4	1	3(279)	RR: 1.51[1.19, 1.90]	0.0	Moderate
Liu, 2014	CHM+ chemotherapy vs. chemotherapy	1	4(339)	OR: 2.60[1.46, 4.63]	29.0	Moderate
*Nasopharyngeal cancer*						
Qiao, 2011	CHM + radiotherapy vs. radiotherapy	3	3(307)	RR: 1.30 [1.03, 1.63]	46.0	Moderate
*Esophageal cancer*						
Du, 2013	KS + radiotherapy alone or KS + chemotherapy + radiotherapy vs. radiotherapy alone or chemotherapy + radiotherapy	3	2(142)	OR: 1.86[0.96, 3.62]	21.4	Low
*Cervical cancer*						
Xu, 2012	CHM+ chemotherapy or radiotherapy vs. chemotherapy or radiotherapy alone	1	4(427)	OR: 4.16[1.97, 8.78]	NR	Moderate
*Various types of cancer*						
Su, 2013	KS+ chemotherapy or radiotherapy vs. chemotherapy or radiotherapy alone	1	9(656)	RR: 1.41[1.23, 1.63]	0.0	Moderate
	KS+ chemotherapy or radiotherapy vs. chemotherapy or radiotherapy alone	2	6(408)	RR: 1.76[1.23, 2.48]	32.7	Moderate

^*^p < 0.05 for the heterogeneity test;

^^^CHM denotes the inclusion of all types of Chinese herbal medicines. The use of generic terms “chemotherapy” or “radiotherapy” denote that the specific treatments used were not described in the original meta-analyses publications.

^¶^Effects on dichotomous data were summarized with risk ratio (RR) or odds ratio (OR) to measure the risk of experiencing certain outcome in the treatment group as compared to the control group.

Keys: CHM, Chinese herbal medicine; CI confidence interval; FOLFOX, the FOLFOX regimen refers to 5-Fluorouracil + Leucovorin combined with Oxaliplatin; KLT, Kanglaite injection; KS, Compound Kushen injection; NR, not reported; OR, odds ratio; RR, relative risk; TACE, Transcatheter arterial chemoembolization.

**Table 5:** Chinese Herbal Medicine for Reducing Chemotherapy Related Toxicity: Overview of Meta-Analyses Results.

First author and year of publication	Cancer cite	Comparison^	Outcome assessment method#	No. of studies (No. of patients)	Pooled results (95%CI)¶	Heterogeneity I^2^ (%)	Quality of evidence
*Leucopenia*							
Wu,2009a	NSCLC	Aidi injection + NP vs. NP	Grade II–IV	13 (1000)	RR: 0.59[0.52, 0.67]	NR	Low
Dong,2010b	NSCLC	SFI+ platinum containing chemotherapy vs. platinum containing chemotherapy	Grade III–IV	20(1643)	RR: 0.37[0.29, 0.47]	0.0	Moderate
Li, 2013	NSCLC	CHM+ chemotherapy vs. chemotherapy	Grade III–IV	9(666)	RR: 0.36[0.26, 0.52]	0.0	Moderate
		CHM+ chemotherapy vs. chemotherapy	Grade I–IV	8(603)	RR: 0.75[0.67, 0.84]	20.0,	Moderate
Xu, 2014	NSCLC	KLT+ chemotherapy vs. chemotherapy	Grade III–IV	10(982)	OR: 0.54[0.38, 0.77]	52.0*	Moderate
Liu,2009	CRC	CHM+ chemotherapy vs. chemotherapy	Grade I	6(334)	RR: 0.50[0.31, 0.80]	7.0	Moderate
		CHM+ chemotherapy vs. chemotherapy	Grade II	6(334)	RR: 0.37[0.21, 0.66]	0.0	Low
		CHM+ chemotherapy vs. chemotherapy	Grade III	5(281)	RR: 0.47[0.19, 1.19]	0.0	Low
		CHM+ chemotherapy vs. chemotherapy	Grade IV	2(129)	RR: 0.13[0.02, 1.05]	0.0	Low
Chen, 2014	CRC	CHM+FOLFOX4 vs. FOLFOX4	Neutropenia grade III–IV	10(692)	RR: 0.33[0.18, 0.60]	0.0	Low
Shi, 2012	Gastric cancer	CHM+ chemotherapy vs. chemotherapy	Grade II–IV	7(353)	OR: 0.26[0.18, 0.37]	35.0	Moderate
Dong, 2010a	Various	Astragalus injection + chemotherapy vs. chemotherapy	Grade I–IV	9(927)	RR: 0.84 [0.79, 0.88]	77.3*	Low
Fu, 2010	Various	CHM+ chemotherapy vs. chemotherapy	Grade I–IV	11(2169)	OR: 0.40 [0.23, 0.68]	55.0*	Moderate
*Nausea and vomiting*							
Wu, 2009a	NSCLC	Aidi injection + NP vs. NP	Grade II–IV	10 (781)	RR: 0.52[0.43, 0.62]	NR	Low
Dong, 2010b	NSCLC	SFI+ platinum containing chemotherapy vs. platinum containing chemotherapy	Grade III–IV	14(1031)	RR: 0.32[0.22, 0.47]	0.0	Moderate
Li, 2013	NSCLC	CHM+ chemotherapy vs. chemotherapy	Grade III–IV	4(295)	RR: 0.24[0.12, 0.50]	0.0	Moderate
Xu, 2014	NSCLC	KLT+ chemotherapy vs. chemotherapy	Grade III–IV	10(982)	OR: 0.54[0.38, 0.77]	52.0*	Moderate
Li, 2012b	Liver cancer	CHM+TACE vs. TACE alone or TACE+ conventional care	Grade I–IV	11(816)	RR: 0.79[0.69, 0.91]	48.0*	Moderate
Cheung, 2013	Liver cancer	CHM+TACE vs. TACE	Grade I–IV	9(581)	RR: 0.86 [0.76, 0.96]	40.0	Moderate
Liu, 2009	CRC	CHM+ chemotherapy vs. chemotherapy	Grade I	6(334)	RR: 0.85 [0.60, 1.20]	0.0	Moderate
		CHM+ chemotherapy vs. chemotherapy	Grade II	6(334)	RR: 0.51 [0.31, 0.84]	0.0	Moderate
		CHM+ chemotherapy vs. chemotherapy	Grade III	6(334)	RR: 0.49 [0.23, 1.05]	0.0	Moderate
		CHM+ chemotherapy vs. chemotherapy	Grade IV	1(61)	RR: 0.11 [0.01, 1.92]	NA	Very low
Chen, 2014	CRC	CHM+FOLFOX4 vs. FOLFOX4	Grade III–IV	9(633)	RR: 0.34[0.18, 0.66]	0.0	Moderate
Shi, 2012	Gastric cancer	CHM+ chemotherapy vs. chemotherapy	Grade II–IV	5(279)	OR: 0.48[0.34, 0.66]	0.0	Moderate
*Thrombocytopenia*							
Dong, 2010b	NSCLC	SFI+ platinum containing chemotherapy vs. platinum containing chemotherapy	Grade III–IV	18(1335)	RR: 0.33[0.21, 0.52]	0.0	Moderate
Li, 2013	NSCLC	CHM+ chemotherapy vs. chemotherapy	Grade III–IV	6(557)	RR: 0.34[0.17, 0.68]	0.0	Moderate
		CHM+ chemotherapy vs. chemotherapy	Grade I–IV	6(494)	RR: 0.43[0.31, 0.60]	0.0	Moderate
Chen, 2014	CRC	CHM+FOLFOX4 vs. FOLFOX4	Grade III–IV	1(42)	RR: 1.00[0.07, 14.95]	NA	Moderate
Shi, 2012	Gastric cancer	CHM+ chemotherapy vs. chemotherapy	Grade II–IV	4(225)	OR: 0.35[0.14, 0.86]	0.0	Moderate
Dong, 2010a	Various	Astragalus + chemotherapy vs. chemotherapy	Grade I–IV	8(829)	RR: 0.69 [0.56, 0.85]	83.5*	Moderate
Fu, 2010	Various	CHM+ chemotherapy vs. chemotherapy	Grade I–IV	7(1162)	OR: 0.41 [0.27, 0.62]	8.9	Moderate
*Anemia*							
Dong, 2010b	NSCLC	SFI+ platinum containing chemotherapy vs. platinum containing chemotherapy	Grade III–IV	15(1161)	RR: 0.44[0.30, 0.66]	0.0	Moderate
Li, 2013	NSCLC	CHM+ chemotherapy vs. chemotherapy	Grade I–IV	6(553)	RR: 0.64[0.51, 0.80]	25.0	Moderate
		CHM+ chemotherapy vs. chemotherapy	Grade III–IV	6(536)	RR: 0.58[0.26, 1.29]	0.0	Moderate
Chen, 2014	CRC	CHM+FOLFOX4 vs. FOLFOX4	Grade III–IV	3(220)	RR: 0.30[0.05, 1.89]	0.0	Low
Shi, 2012	Gastric cancer	CHM+ chemotherapy vs. chemotherapy	Grade II–IV	4(207)	OR: 0.38[0.25, 0.58]	43.0	Moderate
Dong, 2010a	Various	Astragalus+ chemotherapy vs. chemotherapy	Grade I–IV	4(371)	RR: 0.42 [0.27, 0.65]	33.1	Moderate
*Neurotoxicity*							
Liu,2009	CRC	CHM+ chemotherapy vs. chemotherapy	Grade I	5(273)	RR: 0.84 [0.57, 1.24]	0.0	Low
		CHM+ chemotherapy vs. chemotherapy	Grade II	5(273)	RR: 0.73 [0.45, 1.19]	0.0	Low
		CHM+ chemotherapy vs. chemotherapy	Grade III	5(273)	RR: 0.40 [0.13, 1.25]	0.0	Low
Chen, 2014	CRC	CHM+FOLFOX4 vs. FOLFOX4	Grade III–IV	7(529)	RR: 0.39[0.15, 1.00]	0.0	Low
*Other chemotherapy related toxicity*							
Chen, 2014	CRC	CHM+FOLFOX4 vs. FOLFOX4	Diarrhea grade III–IV	5(448)	RR: 0.39[0.11, 1.42]	0.0	Low
		CHM+FOLFOX4 vs. FOLFOX4	Stomatitis grade III–IV	2(210)	RR: 0.43[0.08, 2.31]	0.0	Low

Keys: CHM, Chinese herbal medicine; CI confidence interval; CRC, colorectal cancer; FOLFOX, the FOLFOX regimen refers to 5–Fluorouracil + Leucovorin combined with Oxaliplatin; KLT, Kanglaite injection; NA, not applicable; NSCLC, non-small lung cell cancer; NP, Cisplatin + Vinorelbine; NR, not reported; OR, odds ratio; RR, relative risk; SFI, Shenqi Fuzheng injection; TACE, Transcatheter arterial chemoembolization.

^#^All chemotherapy toxicities were measured with the World Health Organization toxicity criteria;

^*^p < 0.05 for the heterogeneity test;

^^^CHM denotes the inclusion of all types of Chinese herbal medicines. The use of generic terms “chemotherapy” or “radiotherapy” denote that the specific treatments used were not described in the original meta-analyses publications.

^¶^Effects on dichotomous data were summarized with risk ratio (RR) or odds ratio (OR) to measure the risk of experiencing certain outcome in the treatment group as compared to the control group.
