# Supplementary material for: Effectiveness of Chinese herbal medicine for cancer palliative care: overview of systematic reviews with meta-analyses
Source: Sci Rep. 2015 Dec 16;5:18111. doi: 10.1038/srep18111 (PMC4680970; doi:10.1038/srep18111)
Supplement: Appendix 1-4 [file srep18111-s1.pdf]

Effectiveness of Chinese herbal medicine for cancer palliative care: overview of systematic reviews with meta-analysis

Vincent CH Chung[1,2], Xinyin Wu*[1,2], , Edwin P Hui[1,3], Eric TC Ziea[4], Bacon FL Ng[4], Robin ST Ho[2], Kelvin KF Tsoi[2,5], Samuel YS Wong[1,2], Justin CY Wu[1,6]

1. Hong Kong Institute of Integrative Medicine, The Chinese University of Hong Kong, Hong Kong
2. Jockey Club School of Public Health and Primary Care, The Chinese University of Hong Kong, Hong Kong
3. Comprehensive Cancer Trials Unit, The Chinese University of Hong Kong, Hong Kong
4. Chinese Medicine Department, Hong Kong Hospital Authority, Hong Kong
5. Big Data Decision Analytics Research Centre, The Chinese University of Hong Kong, Hong Kong
6. Department of Medicine & Therapeutics, The Chinese University of Hong Kong, Hong Kong

*Corresponding author: Dr. Xinyin Wu, 5/F, School of Public Health Building, Prince of Wales Hospital, Shatin, New Territories, Hong Kong; phone: (+852) 2252 8706; email: wuxinyin@cuhk.edu.hk

Appendix 1. Search strategies and results for meta-analysis on Chinese herbal medicine for cancer palliative care

i) Cochrane Database of Systematic Reviews (CDSR) from inception to 3/9/2014

1	Chinese herb*.mp.	185
2	herb*.mp.	642
3	Traditional Chinese medic*.mp.	229
4	phytother*.mp.	130
5	(chinese adj5 (traditional or medic*)).mp.	452
6	(plant or plants).mp.	347
7	(traditional adj5 medic*).mp.	335
8	Chinese medic*.mp.	348
9	oriental medic*.mp.	26
10	herbaceous agent.mp.	12
11	medicinal plant*.mp.	33
12	1 or 2 or 3 or 4 or 5 or 6 or 7 or 8 or 9 or 10 or 11	1075
13	palliative car*.mp.	165
14	terminal car*.mp.	34
15	terminal ill*.mp.	32
16	palliat*.mp.	452
17	(terminal* and (car* or ill*)).mp.	324
18	((advanced or end stage or terminal*) adj4 (diseas* or ill* or cancer* or malignan*)).mp.	812
19	(last year of life or LYOL or life end or end of life).mp.	95
20	13 or 14 or 15 or 16 or 17 or 18 or 19	1257
**21**	**12 and 20**	**174**

ii) Database of Abstracts of Reviews of Effects (DARE) from inception to 3/9/2014

1	Chinese herb*.mp.	291
2	herb*.mp.	438
3	Traditional Chinese medic*.mp.	71
4	phytother*.mp.	248
5	(chinese adj5 (traditional or medic*)).mp.	294
6	(plant or plants).mp.	311
7	(traditional adj5 medic*).mp.	183
8	Chinese medic*.mp.	136
9	oriental medic*.mp.	19
10	herbaceous agent.mp.	0
11	medicinal plant*.mp.	4
12	1 or 2 or 3 or 4 or 5 or 6 or 7 or 8 or 9 or 10 or 11	804
13	palliative car*.mp.	196
14	terminal car*.mp.	41
15	terminal ill*.mp.	12
16	palliat*.mp.	269
17	(terminal* and (car* or ill*)).mp.	99
18	((advanced or end stage or terminal*) adj4 (diseas* or ill* or cancer* or malignan*)).mp.	555
19	(last year of life or LYOL or life end or end of life).mp.	30
20	13 or 14 or 15 or 16 or 17 or 18 or 19	803
**21**	**12 and 20**	**19**

iii) MEDLINE from inception to 3/9/2014

1	MEDLINE.tw.	54034
2	systematic review.tw.	42879
3	meta analysis.pt.	51267
4	1 or 2 or 3	110972
5	exp palliative care/	41061
6	exp terminal care/	42274
7	exp terminally ill/	5865
8	palliat*.mp.	63977
9	palliative car*.mp.	44119
10	terminal car*.mp.	23513
11	terminally ill*.mp.	9003
12	(terminal* and (car* or ill*)).mp.	37400
13	((advanced or end stage or terminal*) adj4 (diseas* or ill* or cancer* or malignan*)).mp.	118498
14	(last year of life or LYOL or life end or end of life).mp.	11417
15	5 or 6 or 7 or 8 or 9 or 10 or 11 or 12 or 13 or 14	211993
16	exp Drugs, Chinese Herbal/	29347
17	Chinese herb*.mp.	31217
18	exp Medicine, Chinese Traditional/	12454
19	Traditional Chinese medic*.mp.	8905
20	exp Phytotherapy/	30551
21	phytother*.mp.	30659
22	(chinese adj5 (traditional or medic*)).mp.	25236
23	(herbs or herbal).mp.	46181
24	(plant or plants).mp.	496247
25	(traditional adj5 medic*).mp.	38858

26	16 or 17 or 18 or 19 or 20 or 21 or 22 or 23 or 24 or 25	551363
27	**4 and 15 and 26**	46

iv) EMABSE from inception to 3/9/2014

1	meta-analysis.tw.	70115
2	systematic review.tw.	60452
3	1 or 2	111604
4	exp palliative therapy/	66664
5	exp terminal care/	47273
6	exp terminal disease/	4812
7	exp terminally ill patient/	6249
8	exp cancer patient/	126211
9	palliative therap*.mp.	67642
10	terminal car*.mp.	26601
11	terminally ill patient*.mp.	7400
12	cancer patient*.mp.	221822
13	palliat*.mp.	96997
14	(terminal* and (car* or ill*)).mp.	215666
15	((advanced or end stage or terminal*) adj4 (diseas* or ill* or cancer* or malignan*)).mp.	202131
16	(last year of life or LYOL or life end or end of life).mp.	17553
17	4 or 5 or 6 or 7 or 8 or 9 or 10 or 11 or 12 or 13 or 14 or 15 or 16	679148
18	exp Chinese medicine/	24449
19	exp oriental medicine/	2605
20	exp herbaceous agent/	35273
21	exp medicinal plant/	154347
22	exp Chinese herb/	3570
23	Chinese medic*.mp.	40047
24	oriental medic*.mp.	3569

25	herbaceous agent.mp.	35275
26	medicinal plant*.mp.	77812
27	Chinese herb*.mp.	10187
28	herb*.mp.	121347
29	18 or 19 or 20 or 21 or 22 or 23 or 24 or 25 or 26 or 27 or 28 or 29	269392
30	**3 and 17 and 29**	**83**

v) Chinese Biomedical Database (CBM) [Chinese] from inception to 23/7/2014

("系统综述"[全字段] OR "荟萃分析"[全字段] OR "META"[全字段]) AND ("中药"[全字段] OR "中草药"[全字段] OR "中成药"[全字段] OR "中西医"[全字段] OR "中医"[全字段]) AND ("肿瘤"[全字段] OR "癌"[全字段] OR "岩"[全字段]) 277

vi) Wan Fang Digital Journals [Chinese] from inception to 23/7/2014

("系统综述" OR "荟萃分析" OR "META") AND ("中药" OR "中草药" OR "中成药" OR "中西医" OR "中医") AND ("肿瘤" OR "癌" OR "岩") 209

vii) Taiwan Periodical Literature Databases [Chinese] from inception to 23/7/2014

(TX=系統綜述 OR 薈萃分析 OR META) [AND] (TX=中藥 OR 中草藥 OR 中成藥 OR 中西醫 OR 中醫) [AND] (TX=腫瘤 OR 癌 OR 岩) 36 none included

Appendix 2. Detailed operational guide for applying the AMSTAR tool*

**Amstar 1. Was an 'a priori' design provided?**<br>The research question and inclusion criteria should be established before the conduct of the review.<br>**Operational Definition (OD):** A “Yes” will be given if the review has published a protocol for the systematic review.	❒ Yes<br>❒ No
**Amstar 2. Was there duplicate study selection and data extraction?**<br>There should be at least two independent data extractors and a consensus procedure for disagreements should be in place.<br>**OD:** A “Yes” will be given if:<br>**(i)** Two reviewers performed study selection, AND<br>**(ii)** Two reviewers performed data extraction AND<br>**(iii)** Consensus process was implemented for resolving disagreement.	❒ Yes (i + ii + iii)<br>❒ No (These 3 criteria were not fulfilled)<br>❒ Could not answer (i.e. not reported)
**Amstar 3. Was a comprehensive literature search performed?**<br>At least two electronic sources should be searched. The report must include years and databases used (e.g., CENTRAL, EMBASE, and MEDLINE). Key words and/or MESH terms must be stated and where feasible the search strategy should be provided. All searches should be supplemented by consulting current contents, reviews, textbooks, specialized registers, or experts in the particular field of study, and by reviewing the references in the studies found.<br>**OD:** A “Yes” will be given if at least two electronic sources plus one supplementary strategy were used (e.g. Cochrane register/CENTRAL counts as two sources; a grey literature search counts as supplementary). (SIGLE database, dissertations, conference proceedings, and trial registries are all considered grey)	❒ Yes<br>❒ No<br>❒ Could not answer (i.e. not reported)
**Amstar 4. Was the status of publication (i.e. grey literature) used as an inclusion criterion?**<br>The authors should state that they searched for reports regardless of their publication type. The authors should state whether or not they excluded any reports from the systematic review, based on their publication status, language etc.<br>If review indicates that there was a search for “grey literature” or “unpublished literature,” indicate “yes.” SIGLE database, dissertations, conference proceedings, and trial registries are all considered grey for this purpose. If searching a source that contains both grey and non-grey, must specify that they were searching for grey/unpublished literature.<br>**OD:** If eligibility criterion is restricted to “non-grey” literature, a “No” will be given, which indicates a methodological shortcoming in this domain.	❒ Yes<br>❒ No<br>❒ Could not answer (i.e. not reported)
**Amstar 5. Was a list of studies (included and excluded) provided?**<br>A list of included and excluded studies should be provided.<br>**OD:** A “Yes” will be given if the included and excluded studies are referenced.	❒ Yes<br>❒ No
**Amstar 6. Were the characteristics of the included studies provided?**<br>In an aggregated form such as a table, data from the original studies should be provided on the participants, interventions and outcomes. The ranges of characteristics in all the studies analyzed e.g., age, race, sex, relevant	❒ Yes<br>❒ No

socioeconomic data, disease status, duration, severity, or other diseases should be reported.<br>**OD:** A “Yes” will be given if the information described above is presented appropriately.	
**Amstar 7. Was the scientific quality of the included studies assessed and documented?**<br>'A priori' methods of assessment should be provided (e.g., the use of Cochrane Risk of Bias tool as a mean to assessment); for other types of studies alternative tools will also be acceptable.<br>**OD:** To score a “Yes”, the authors should report risk of bias level in each of the methodological domain included in the risk of bias assessment tool that the authors have chosen to use.	❒ Yes<br>❒ No
**Amstar 8. Was the scientific quality of the included studies used appropriately in formulating conclusions?**<br>The results of the methodological rigor and scientific quality should be considered in the analysis and the conclusions of the review, and *considerations on how risk of bias among included study may impact conclusion should be explicitly stated*.<br>**OD:** To score a “Yes”, the reviewers must consider risk of bias explicitly when writing the conclusion section of the MA. For example, a reviewer may state: the results should be interpreted with caution due to high risk of bias among included studies. A “No” will be given if answer to Q7 is “No”.	❒ Yes<br>❒ No
**Amstar 9. Were the methods used to combine the findings of studies appropriate?**<br>For meta-analysis, statistical tests should be done to ensure that the studies were combinable by assessing their homogeneity. This can be done by using the Cochran Q test or reporting the I^2 value.<br>**OD:** “Yes” will be given when one of the two situation applies:<br>(i) Homogeneity is found, and authors used fixed effect model or random effect model, or<br>(ii) Heterogeneity is found, and authors performed appropriate subgroup analysis or meta-regression.<br><br>“No” will be given when one of the two situation applies:<br>(i) Heterogeneity is found, and authors used fixed or random effect model and reported the results directly without highlighting the role of heterogeneity;<br>(ii) Heterogeneity is not assessed or reported.	❒ Yes<br>❒ No
**Amstar 10. Was the likelihood of publication bias assessed?**<br>An assessment of publication bias should include a combination of graphical aids (e.g., funnel plot) and/or statistical tests (e.g., Egger regression test).<br>**OD:** A “No” will be given if no relevant test values or funnel plot was reported. However, a “Yes” will still be given if authors mentioned that publication bias could not be assessed because there were fewer than 10 included studies [1].	❒ Yes<br>❒ No
**Amstar 11. Was the conflict of interest included?**<br>Potential sources of support should be clearly acknowledged in both the	

systematic review and the included studies.<br>**OD:** To score a "Yes", the authors must indicate source of funding or support for the systematic review AND for each of the included studies. A "Yes" will still be scored if the authors acknowledged that funding sources for included randomized controlled trials were unknown.	❒ Yes<br>❒ No

*adapted from the official AMSTAR website (http://www.amstar.ca/)

Appendix 3. Lists of included studies

1 Cui, H.-J., He, H.-Y. & Lin, Y. A Systematic Review for compound Kushen injection combined with NP model chemotherapy for the treatment of advanced stage of non-small cell lung cancer [Chinese]. *Anti-tumor Pharmacy* **1**, 72-77 (2011).

2 Ma, Y.-L. & Chen, L.-L. Systematic review on Yanshu injection plus NP for terminal NSCLC [Chinese]. *CHIN J CANCER PREV TREAT* **18**, 1611-1615 (2011).

3 Zhu, L.-N., Yang, Z.-J., Wang, S.-Y. & Tang, Y.-M. Kanglaite for Treating Advanced Non-small-cell Lung Cancer: A Systematic Review [Chinese]. *Chin J Lung Cancer* **12**, 208-215 (2009).

4 Chen, W., Shi, J., Li, J.-C. & Pan, Q. Meta-analysis on Integrated Chinese-Western Therapy in Non-small Cell Lung Cancer [Chinese]. *Practical Preventive Medicine* **17**, 1360-1363, doi:10.3969/j.issn.1006-3110.2010.07.048 (2010).

5 Dong, J., Lv, H.-Y. & Mao, J.-E. The Enhancing Efficient and Reducing Toxicity of Astragalus in Tumor Chemotherapy: A Meta-analysis [Chinese]. *Guiding Journal of Traditional Chinese Medicine and Pharmcy* **16**, 87-92, doi:10.3969/j.issn.1672-951X.2010.03.051 (2010).

6 Fu, J., Yu, J., Xu, H.-B. & Li, L. Systematic evaluation about efficiency detoxification of Chinese traditional medicine adjuvant chemotherapy for solid tumors [Chinese]. *Guiding Journal of Traditional Chinese Medicine and Pharmcy* **16**, 108-112, doi:10.3969/j.issn.1672-951X.2010.10.058 (2010).

7 Guo, Y.-L., Zhong, S., Hu, X.-Y. & Tu, X. Herbal Therapy of Strengthening Healthy Qi and Expelling Evils Combined with TACE on the Life Quality Improvement in the Patient at the Middle and Late Stage of Primary Liver Cancer： a Systematic Review [Chinese]. *Research of Intergrated Traditional Chinese and Western Medicine* **2**, 116-121, doi:10.3870/j.issn.1674-4616.2010.03.002 (2010).

8 Zhou, A.-G., Li, Y., Hong, S., Mao, X.-M. & Dong, J.-R. Meta-analysis on the effectiveness of Chinese Medicine plus Chemotherapy for the treatment of gastric cancer [Chinese]. *World Journal of Integrated Traditional and Western Medicine* **5**, 376-381, doi:10.3969/j.issn.1673-6613.2010.05.004 (2010).

9 Dong, J., Su, S. Y., Wang, M. Y. & Zhan, Z. Shenqi fuzheng, an injection concocted from chinese medicinal herbs, combined with platinum-based chemotherapy for advanced non-small cell lung cancer: A systematic review. *Journal of Experimental and Clinical Cancer Research* **29** (2010).

10 Liu, L., Wu, W.-J. & Zhou, Z.-H. Meta-analysis of Compound Kuseng Injection plus Chemotherapy for Short Time Effectiveness and Quality of Life on Cancer Patients [Chinese]. *Journal of Zhejiang Chinese Medicine* **47**, 297-299, doi:10.3969/j.issn.0411-8421.2012.04.054 (2012).

11 Fan, Z.-Z., Wang, X., Wang, Y.-J., Jiang, J. & Tian, J.-H. A Meta Analysis of Yanshu Injeetion Plus Chemotherapy for Breast Cancer [Chinese]. *JoURNAL OF LIAONING UNⅣERSITY oF TCM* **14**, 3 pages (2012).

12 Qiao, B. *et al.* Protective Effects of Decoction Plus Radiotherapy on Nasopharyngeal Carcinoma： A Meta-analysis of Randomized Controlled Trial [Chinese]. *West China Medical Journal* **26**, 391-398 (2011).

13 Wang, Z.-Q. & Wang, M.-X. Meta-analysis of Xiaoaiping Injection for the treatment of advanced malignanlies [Chinese]. *Pharmaceutical and Clinical Research* **20**, 531-534, doi:10.3969/j.issn.1673-

7806.2012.06.013 (2012).
14 Zhang, S., Zhang, L. & Shang, H.-C. Systematic Review on Efficacy Enhancing and Toxicity Reducing Functions of Shengifuzheng Injection in Patients with Malignant Tumors [Chinese]. *China Licensed Pharmacist* **9**, 17-24, doi:10.3969/j.issn.1672-5433.2012.12.005 (2012).
15 Rong, Z., Wei, H.-X. & Huang, H.-F. Traditional Chinese Medicine in Combination with Chemotherapy for Senile Mid-advanced Non-small Cell Lung Cancer: A Systematic Review [Chinese]. *JOURNAL OF LIAONING UNIVERSITY OF TCM* **34**, 1061-1065 (2012).
16 Li, S. G. *et al.* The efficacy of Chinese herbal medicine as an adjunctive therapy for advanced non-small cell lung cancer: a systematic review and meta-analysis. *PLoS ONE [Electronic Resource]* **8**, e57604 (2013).
17 Tian, X., Zhou, F.-X., Wang, W.-G. & Jia, L.-Q. M eta-Analysis of Integrative Therapy for Small Cell Lung Cancer [Chinese]. *Journal of Traditional Chinese Medicine* **54**, 927-930 (2013).
18 Wu, P., Dugoua, J. J., Eyawo, O. & Mills, E. J. Traditional Chinese Medicines in the treatment of hepatocellular cancers: a systematic review and meta-analysis. *Journal of experimental & clinical cancer research : CR* **28**, 112, doi:10.1186/1756-9966-28-112 (2009).
19 Ma, Y.-L. & Chen, L.-L. Systematic Review on Yanshu Injection Plus TACE for Primary Liver Cancer [Chinese]. *Chinese Journal of Chinese Medicine Phamacy information* **18**, 29-31, doi:10.3969/j.issn.1005-5304.2011.07.011 (2011).
20 Li, X.-Q. & Ling, C.-Q. Chinese herbal medicine for side effects of transarteriaI chemoembolization in liver cancer patients： a systematic review and meta-analysis [Chinese]. *Journal of Chinese Intergrative Medicine* **10**, 1341-1362, doi:10.3736/jcim20121204 (2012).
21 Cheung, F. *et al.* Chinese medicines as an adjuvant therapy for unresectable hepatocellular carcinoma during transarterial chemoembolization: A meta-analysis of randomized controlled trials. *Evidence-based Complementary and Alternative Medicine* **2013** (2013).
22 Jiang, S.-L., Liu, R. & Hua, B.-J. Systematic Review of TCM Com bined with TACE in Treatm ent for Prim ary Liver Carcinoma [Chinese]. *Journal of Liaoning Traditional Chinese Medicine* **40**, 2406-2409 (2013).
23 Wang, C., Wang, Q., He, X.-R., Pan, W. & Guan, Q.-L. Meta-analysis on clinical curative effect of KLT plus Chemotherapy in treatment of advanced gastric cancer [Chinese]. *Modern Journal of Integrated Traditional Chinese and Western Medicin* **20**, 3774-3777 (2011).
24 Xie, X. *et al.* Efficacy and safety of Huachansu combined with chemotherapy in advanced gastric cancer: a meta-analysis. *Medical Hypotheses* **81**, 243-250 (2013).
25 Shi, G.-J., Shan, G.-Z., Zhou, Y.-Q. & Yang, H.-F. Meta-Analysis of Traditional Chinese Medicine Plus Chemotherapy in Treatment of Postoperative Gastric Cancer [Chinese]. *Chinese Journal of Experimental Traditional Medical Formulae* **18**, 261-266, doi:10.3969/j.issn.1005-9903.2012.01.071 (2012).
26 Guo, Z., Jia, X., Liu, J. P., Liao, J. & Yang, Y. Herbal medicines for advanced colorectal cancer. *Cochrane Database of Systematic Reviews* **5**, CD004653 (2012).

27 Li, C.-H., Fu, G.-C., Yang, Y., Chen, Y.-J. & Jing, L.-L. Meta-analysis of Intergrative Medicine on Survival Time in Patients with Advanced Colorectal Cancer [Chinese]. *Journal of Mathematical Medicine* **25**, 520-525, doi:10.3969/j.issn.1004-4337.2012.05.007 (2012).

28 Chen, M., May, B. H., Zhou, I. W., Xue, C. C. & Zhang, A. L. FOLFOX 4 combined with herbal medicine for advanced colorectal cancer: a systematic review. *Phytotherapy research : PTR* **28**, 976-991, doi:10.1002/ptr.5092 (2014).

29 Liu, L.-R. & Zhu, Y.-J. Meta-analysis on Efficacy and Life Quality of Advanced Colorectal Cancer with Chinese Medicine Combined with Chemotherapy [Chinese]. *Journal of Nanjing University of TCM* **30**, 3 (2014).

30 Wang, B., Zhou, M., Li, J. & Chen, D. Meta analysis on therapeutic effects of treating advanced gastric cancer with TCM combined with western medicine [Chinese]. *Int J Trad Chin Med* **34**, 5, doi:10.3760/cma.j.issn.1673-4246.2012.12.002 (2012).

31 Du, Y.-Q., Xie, D., Yang, G.-J. & Ha, M.-L.-T. Clinical Efficacy of Compound Kushen Injection Combined with Radiotherapy in the Treatment of Esophageal Carcinoma: a Meta Analysis [Chinese]. *Anti-tumor Pharmacy*, 7, doi:10.3969/j.issn.2095-1264.2013.097 (2013).

32 Xu, M. *et al.* Adjuvant phytotherapy in the treatment of cervical cancer: a systematic review and meta-analysis. *Journal of alternative and complementary medicine (New York, N.Y.)* **15**, 1347-1353, doi:10.1089/acm.2009.0202 (2009).

33 Su, R., Li, L., Xu, H.-B. & Huang, F. A Systematic Review of Therapeutic Efficacy and Safety of Adjuvant Therapy of Compound Sophora flavescens Injection in the Treatment of Tumor [Chinese]. *China Pharmacy* **24**, 4154-4163 (2013).

34 Xu, X.-W., Lin, G.-Y., Yuan, Z.-Z. & Wang, X.-K. System Review on Kanglaite Combined with Chemotherapy in Treatment of Non-Small Cell Lung Cancer [Chinese]. *CHINESE ARCHIVES OF TRADITIONAL CHINESE MEDICINE* **32**, 733-739 (2014).

35 Wang, X.-F., Tan, S.-Y. & Guo, Y. Adjuvant Treatment of Gastric Cancer Patients with Shenqi Fuzheng Injection： A Systematic Review [Chinese]. *JOURNAL OF LIAONING UNIVERSITY OF TCM* **20**, 126-127 (2014).

36 Liu, J. & Zhu, Q. The Reduction of Adverse Drug Reaction Incidences of Colorectal Caner Patients Receiving Jianpi Herbs Combined with Chemotherapy: A Systematic Review[Chinese]. *Chin J Evid-based Med* **9**, 802-808, doi:10.3969/j.issn.1672-2531.2009.07.013 (2009).

37 Wu, B.-C., Xu, L. & Chen, M. Meta-analysis of Aidi injection plus NP treatment for advanced non-small cell lung cancer [Chinese]. *Zhejiang Journal of Intergrated Traditional Chinese and Western Medicine* **19**, 446-447 (2009).

38 Ma, L., Weng, Y.-N. & Xiao, X. Treatment of NSCLC by tratidional medicine and chemotherapy [Chinese]. *CHINESE JOURNAL OF THE PRACTICAL CHINESE WITH MODERN MEDICINE* **17**, 709-712 (2004).

39 Qin, Y.-F., Ji, H.-L. & Liu, Y.-Y. Systematic reviews of Kangai for treatment of non—small cell Injection together with chemotherapy lung cancer [Chinese]. *CHIN J CANCER PREV TREAT* **19**, 921-924 (2012).

40 Wei, Z., Li, L., Zhan, T. & Lin, G.-Q. Meta-analysis of Kang Ai Injeetion Together with TP Model Chemotherapy for Treatment of Non-smalI Cell Lung Cance [Chinese]. *Chinese Journa1 of Informat ion on TCM* **18**, 29-32, doi:10.3969/j.issn.1005-5304.2011.05.011 (2011).
41 Jin, X., Julieta, R. B., lSze, M. D. & Chan, G. C. F. Ganoderma lucidum (Reishi mushroom) for cancer treatment. *Cochrane Database Syst Rev.*, CD007731 (2012).
42 Yang, S. *et al.* Meta-analysis of the effectiveness of Chinese and Western integrative medicine on medium and advanced lung cancer. *Chinese Journal of Integrative Medicine* **18**, 862-867 (2012).
43 Cai, Y.-N. Efficacy of shenqi fuzheng injection for stomach cancer： a systematic review [Chinese]. *Pharmacy and Clinics of Chinese Materia Medica* **3**, 25-27 (2012).
44 WANG, Y., SHEN, M.-H. & LIN, S.-Y. Meta-analysis of Integration of Traditional and W estern Medicine Treatment Versus W estern Treatment in Patients with ColorectaI Cancer [Chinese]. *CHINESE ARCHIVES OF TRADlTIONAL CHINESE MEDICINE* **14**, 42-44 (2012).
45 Ma, Y.-L. & Chen, L.-L. Meta-analysis on compound kushen injection plus TP chemotherapy for advanced non-small cell lung cancer [Chinese]. *Zhejiang Journal of Intergrated Traditional Chinese and Western Medicine* **22**, 828-831 (2012).
46 Liu, C.-X. *et al.* Zilongjin for NSCLC： A Systematic Review [Chinese]. *Journal of Liaoning Traditional Chinese Medicine* **40**, 2448-2453 (2013).
47 Yang, X., Wang, Y. & Wang, W.-Y. Meta-analysis of traditional Chinese medicine combined with chemotherapy in postoperative patients with gastric cancer. *China Medical Herald* **10**, 67-70 (2013).
48 Xiao, N. & Zhou, X.-M. Meta-analysis on treatment of non-small cell lung cancer with Xiaoaiping injection in combination with platinum-contained first-line chemotherapy [Chinese]. *Modern Journal of Integrated Traditional Chinese and Western Medicine* **22**, 6 (2013).
49 Yan, X.-L. & Gu, Y.-H. Meta-analysis of Kanglaite Injection combined with NP Regimen Chemtherapy for Advanced Non-small Cell Lung Cancer [Chinese]. *Chinese General Practice* **16**, 431-435 (2013).
50 Sheng, L., Fang, Y.-L., Xu, M. & Li, Y. Shenqi Fuzheng injection adjuvant chemotherapy for advanced non—small cell lung cancer： a systematic review [Chinese]. *Chin J New Drugs Clin Rem* **32**, 1-9 (2013).
51 He, H.-L. *et al.* Meta-analysis on treatement of non-small cell lung cancer with shenfu injection in combination with platinum-contained first-line chemotherapy [Chinese]. *Chinese Journal of Experimental Traditional Medical Formulae* **19**, 331-339 (2013).

Appendix 4. Details of CHM evaluated in included meta-analyses

Author and year of publication	Details of CHM
Ma, 2004	Kanglaite injection, Huangqi, Baihua Sheshe herbal, Yifei Baidu decoction, Guben Yiliu II, Yifei Baidu decoction
Liu,2009	Jianpi CHM, Jianpi Xiaoji decoction, Wenshen Jianpi formula, Jianpi Huoxue CHM, Xiaoliu formula, Yiqi Huoxue CHM
Wu, 2009b	Shentao Ruangan Bolus, Aiyishu injection, Hugan Ruanjian formula, Chinese toad bufotoxin injection, Chinese herbal compound (no details), Chinese toad bufotoxin injection, Shenqi capsule, Jew Ear Parasitized Granula, modified six nobles decoction decoction, Gan Ji grain, Ai Di injection, Delishen injection, compound Kushen injection, pingxiao capsule, Kang lai Te injection, AC-III injection, Peiben Guyuan anti-cancer capsule, Yiganjian, pingxiao capsule, Ganfu Kang Capsule, Jinlong capsule, Fuzhenhuaji detoxification pill, Shanxian Granula, Qining injection, Qinggan Jiedu Sanjie decoction, Kang Ai injection, Kanglaite capsule, Xiao Yao San, Qinggan Huayu oral liquid, Fuzhen detoxification decoction.
Chen, 2010	No details on CHM provided.
Fu,2010	No details on CHM provided.
Guo,2010	Included RCTs used Huangqi, Dangshen, Baishu, Shengdi, Gouqi, Nvzhenzi, Wuguteng, Baomao, Baihua Sheshe herbal, Qian herbal, Kushen, Funing, Sanling, Eshu.
Zhou,2010	Sheqi Fuzhen injection, Fuzhen anti-cancer granules, Qingyu Fuzheng tecoction, Jianpi Yiwei granules, Pingxiao capsule, Chinese herbal compound, Weining granule, Fuzheng Quxie decoction, Fuzheng Hewei mixture, Fuzheng Huoxue anti-cancer formula, Shengxue decoction, Chinese Medicine Weichang An.
Qin, 2012a	Yiqi Xiaoji formula , Jiangni Ling, Jianpi Jiedu formula, Yiqi Huoxue Buchang decoction, Quxie capsule, Yiqi Huoxue Chinese herbal medicines, Xiaozheng formula, Sijunzi decoction, Xuefu Zhuyu decoction, herbal medicines based on syndrome differentiation, Yiqi Jiedu decoction, Fructus Brucea Oil Emulsion injection, Kangai injection, Delisheng injection, Shengmai injection, Aidi injection.
Yang, 2012	Compound Kushen injection, Lung Nourishing Antitumor Beverage, Qiankun capsule, Antitumor Beverage, Dialectic treating method of Chinese Medicine, Asparagus Granule, Shenmai injection, Kang Ai injection, Xiaoliu formula.
Wang, 2012a	Self-developed Guben anti-cancer decoction, Fuzheng Huayu Jiedu Sanjie Chinese Medicine, Weishi Shenning Baishu decoction Jiajian, Changfukang capsule, Wenshen Jianpi formula, self-developed formula (no details), Jianpi anti-cancer formula, Jianpi Jiandu formula.
Li, 2012a	Fuzheng anti-cancer decoction, Tianma granules, Jiawei Shengxue decoction, Yiqi Huoxue Chinese Medicine, Bianzheng Treatment, Jianpi Xiaoai Beverage, Quxie capsule, Fuzheng anti-cancer formula, Guben Xiaoai capusle, Jianpi Huashi formula, Yiqi Jiedu decoction, Yiqi Tiaofu decoction, Anti-cancer fangyi pill, Changan formula.

Li, 2012b	Zini Yigan formula, Huolisu oral liquid, Dikang Lishu, panaxoside capsules, Huaier Granule, CHM (no details), Jinlong capsule, Matrine injection, Sianpi Huoxue formula, Jianpi Huoxue formula, Cinobufacini injection, Fuzheng Jiedu formula, Qinggan Jiedu Sanjie decoction, Kang AI injection, Huaji Jianpi formula, Jianpi Jiedu formula, Qingkailing injection, Chaishao Liujunzi decoction, Yigan Decoction, Kangganai complex mixtrure, Jianpi Jiedu Sanjie decoction, Jianpi Shugan decoction, Yiqi Huoxue CHM, Bushen Jianpi formula, Qinggan Huayu oral liquid, Guben Quxie CHM, Xiaoyao San, anti-cancer formula, Huqi san, Sanhuang Sanjie granule, Hugan Xiaozhen decoction, Sanjie Xiaotong Paste, Yangzheng Xiaoji capsule, Huoxue Baoxue CHM, Rougan Jianpi formula.
Shi, 2012	Banxie Xiexin decocion Jiawei, Bushen Jianpi Xiaozheng formula, Tiaobu Qixue Chinese Medicine, Jianpi Bushen formula, Jianpi Huoxue Jiedu formula, Jianwei Yingxiao decoction, Shengmai injection, Sixiao Shejia pill, Xiangsha Liujunzi pill, Yiqi Bushen oral liquid, Yiqi Jianpi, Weichangan Chinese Medicine, Fuyuan Hezhong decoction, Jianpi Yiqi Yangxue, Qianpi Hewei Chinese Medicine, Bushen Jianpi Xiaozheng formula, Bupi Yishen decoction, Jianpi Bushen decoction, Jianpi Keaining based Bianzheng Lunzhi, Jianpi Xiaojiying, Shenbi Jianzhong decoction.
Wang, 2012b	No details on CHM provided.
Rong, 2012	Bushen Tianjing Chinese Medicine, Shenfu injection, Shenqi Fuzheng injection, Lanxiang Xiru injection, Kanglaite injection, Kang Ai injection, Compounds Keshen injection, Fuzheng Xiaoai I, Yadanzi Youru injection, CHM (no details), Bazheng decoction, Hechan pill, ShenYi capsule, Bianzhen Lunzhi, Kangsaidi capsule.
Xu, 2012	No details on CHM provided.
Cheung, 2013	Ai Di injection, Kang Ai injection, Gangfukang capsule, Fuzheng Pinggan Xiaoliu decoction, Jing Long capsule, Blood-activating and stasis-resolving herbs, Kanglaite injection, Ci Dan capsule, Brucea javanica oil injection, CHM for fortifying the spleen and activating the blood, Fuzheng Kang Ai decoction, Matrine injection, Buzhong Yiqi decoction, Yanggan Kang Ai pill, Tanreqing injection, Aitongxiao granule, Huachansu injection, Fugan injection, Fuzheng Jiedu decoction, Aiyishu injection, 960 mixture, Compound Kusheng injection, Ganji granule, Jianpi Qinggan Heji, Qishu Formula, CHM (no details), Yiguan Jianjia Wei, Huagan Ruanjian formula, CHM for fortifying the spleen and resolving dampness and activating the blood and detoxifying, Sijunzi decoction, Lianhua Qinggan Yin, CHM for soothing the liver, fortifying the spleen, and tonifying the kidney, Hugan Xiaozheng decoction, Sanjie Xiaotong Gao, Chaishao Liujunzi decoction, Jianpi Hugan decoction, Guben Yiliu II, Canqi capsule.
Li, 2013	Shenmai injection, gujin grand decoction, Feiji recipe, Feitai capsule, Yinqi Yangyin decoction, Ziyin Qinre Jiedu decoction, Jiapi Yangxue decoction, Xiaoji decoction, Haishensu, Fuzhenjiedu decoction, Hechan Pian, compounds Kushen injection, CHM decoction (no detail), Kangliu Zengxiao decoction, Feiyan Ning decoction, Meihua Dianshe pill, Yangyin Ruanjian decoction, Artesunate, Feiliuping extract, Feiliuping decoction, Hechan Pian, Shenyi capsule, Yiqi Yangyin Huatan decoction.
Tian, 2013	Yiqi Qingfei capsule, Shenqi Fuzheng injection, Sanyi capsule, compound Kushen injection, compound Zaofan pill, Bianzheng Lunzhi, Qingfei capsule, Kangfuxin liquid, Peiben Yijin decoction, Xiaobanxia decoction, Kang Ai injection, Shenling Baishu San granule, Xidan decoction.
Jiang, 2013	No details on CHM provided.
Xu, 2013	No details on CHM provided.

Chen, 2014	Compound Kushen injection, Javanica oil Emulsion Injection, Aidi Injection, Wenshen Jianpi decoction, Kang Ai Injection, Fupi Yiwei decoction, Jiangniling decoction, Ginsenoside Rg3 capsules, 3 CHM decoctions based on CHM ‘Zheng’ differentiation, Guben Xiaoliu Capsule, Jianpi Jiedu decoction.
Liu, 2014	No details on CHM provided.

Keys: CHM, Chinese herbal medicine.
